# A universal mechanism generating clusters of differentiated loci during divergence‐with‐migration

**DOI:** 10.1111/evo.12957

**Published:** 2016-06-01

**Authors:** Marina Rafajlović, Anna Emanuelsson, Kerstin Johannesson, Roger K. Butlin, Bernhard Mehlig

**Affiliations:** ^1^ Department of PhysicsUniversity of GothenburgSE‐412 96GothenburgSweden; ^2^ The Linnaeus Centre for Marine Evolutionary BiologyUniversity of GothenburgSE‐405 30GothenburgSweden; ^3^ Department of Marine Sciences—TjärnöUniversity of GothenburgSE‐452 96StrömstadSweden; ^4^ Department of Animal and Plant SciencesUniversity of SheffieldSheffield S10 2TNUnited Kingdom

**Keywords:** Concentrated genetic architecture, divergence hitchhiking, divergent selection, genomic hitchhiking, islands of divergence, stochastic loss

## Abstract

Genome‐wide patterns of genetic divergence reveal mechanisms of adaptation under gene flow. Empirical data show that divergence is mostly concentrated in narrow genomic regions. This pattern may arise because differentiated loci protect nearby mutations from gene flow, but recent theory suggests this mechanism is insufficient to explain the emergence of concentrated differentiation during biologically realistic timescales. Critically, earlier theory neglects an inevitable consequence of genetic drift: stochastic loss of local genomic divergence. Here, we demonstrate that the rate of stochastic loss of weak local differentiation increases with recombination distance to a strongly diverged locus and, above a critical recombination distance, local loss is faster than local “gain” of new differentiation. Under high migration and weak selection, this critical recombination distance is much smaller than the total recombination distance of the genomic region under selection. Consequently, divergence between populations increases by net gain of new differentiation within the critical recombination distance, resulting in tightly linked clusters of divergence. The mechanism responsible is the balance between stochastic loss and gain of weak local differentiation, a mechanism acting universally throughout the genome. Our results will help to explain empirical observations and lead to novel predictions regarding changes in genomic architectures during adaptive divergence.

In spite of substantial gene flow, populations under differential selection in a heterogeneous environment may diverge as partial barriers to gene exchange establish along the genome at loci involved in local adaptation (Barton and Bengtsson 1986). If the combined effects of these barriers are strong enough, gene flow may eventually cease and result in ecological speciation (Nosil 2012; Flaxman et al. 2013). Local adaptation of populations is observed everywhere in nature (Savolainen et al. 2013), but the genetic mechanisms involved at various stages of differentiation remain poorly understood. In particular, it is not known what mechanisms allow populations under differential selection and gene flow to diverge and, potentially, evolve into distinct species (Seehausen et al. 2014). This may depend, in part, on the genomic architecture of adaptive divergence (Smadja and Butlin 2011). Genome scans reveal that different species have very different numbers of loci that cause traits to diverge, ranging from one or a few loci of large effect to hundreds of loci each with presumably smaller effect (Seehausen et al. 2014; Marques et al. 2016). A very intriguing empirical observation is that loci exhibiting divergence may not be uniformly distributed across the genome (Via 2009; Feder et al. 2012, 2013; Seehausen et al. 2014). Instead “islands of divergence” or “clustered genetic architectures” are commonly observed (Feder et al. 2012, 2013; Jones et al. 2012; Marques et al. 2016), while there are few examples of divergent ecotypes in which observed genetic differentiation appears homogeneous (reviewed by Feder et al. 2013, but see Soria‐Carrasco et al. 2014). Unveiling the mechanisms involved in establishing a nonuniform distribution of divergent loci is a key step toward understanding both local adaptation and speciation under gene flow.

Gene flow due to migration between populations subject to divergent selection opposes differentiation. However, if divergence is established at one or a few loci, the effective migration rate in the genomic regions surrounding these loci is reduced due to linkage (Bengtsson 1985; Barton and Bengtsson 1986). For an illustration of this effect in infinitely large populations see, for example, Figure 3B in Barton and Bengtsson (1986) and Figure 1 in Feder and Nosil (2010; note that Feder and Nosil 2010 simulated populations without drift, varying population size only to infer effects of gene flow on levels of differentiation). An instructive interpretation of these results is that the effect of indirect selection (the source of which is a diverged locus) weakens as the recombination distance from this locus increases. By contrast to infinitely large populations, this linkage to a diverged locus has two consequences in populations of finite size where random genetic drift is necessarily at work. First, the establishment probability of a new beneficial mutation is higher for the mutation landing closer to an already diverged locus than further away (hereafter, *the establishment bias*). For an illustration of this effect, see Figure 3 in Feder et al. (2012). Second, genetic drift may result in loss of differentiation if, by chance, the same allele becomes fixed in both diverging populations. This effect may be opposed by linkage to another differentiated genomic region under divergent selection. As a consequence, the rate of stochastic loss of differentiation may be larger at larger recombination distances from another diverged genomic region (see Aeschbacher and Bürger 2014 for an analysis of this effect in a mainland–island model of divergence). Both the establishment‐bias and the stochastic‐loss effect necessarily influence the genetic patterns emerging during divergence‐with‐migration. Yet, earlier theoretical studies have focused only on understanding the importance of the establishment‐bias effect (Yeaman and Whitlock 2011; Feder et al. 2012, 2013; Yeaman 2013). Disregarding stochastic loss has, for example, led Feder et al. (2012) to conclude that clustering of differentiated loci may occur only during early stages of divergence‐with‐migration because this is when the establishment bias is strong. These authors further conclude that, as divergence is ongoing, the establishment of new mutations becomes facilitated over the whole genome, and the establishment bias inevitably weakens (see Figs. 5D–F and 6 in Feder et al. 2012). Consequently, these authors predict that clusters of differentiated loci disappear during late stages of divergence and, instead, genome‐wide, uniformly distributed differentiation appears (Feder et al. 2012). This effect is referred to as *genome hitchhiking* by Feder et al. (2012). However, this genome‐hitchhiking prediction is difficult to reconcile with earlier results of multilocus simulations of divergence‐with‐migration (Yeaman and Whitlock 2011), showing that divergence patterns increasingly concentrate during the late stages of divergence. Furthermore, in a later study Yeaman (2013) finds that the establishment bias is not important when many loci underlie a selected trait, and this is true even during the early stages of divergence (for which Feder et al. 2012 found that the establishment bias is strongest). This is because during the early stages of divergence, only a few loci manage to differentiate, and the probability that a mutation lands near any one of these few initially diverged loci is much smaller than the probability that it lands anywhere else in the genome. Based on this result, and disregarding stochastic loss, Yeaman (2013) concludes that clusters of differentiated loci cannot emerge in natural populations during biologically realistic timescales unless other mechanisms are invoked that suppress recombination, such as genomic rearrangements.

**Figure 1:**
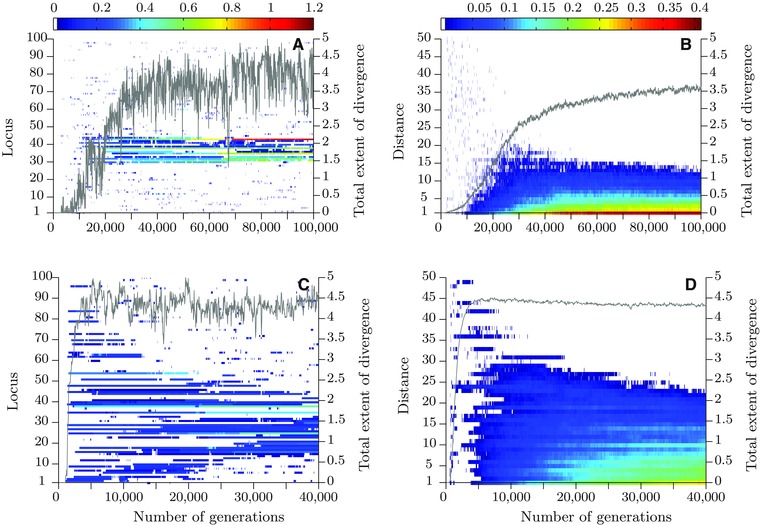
Multilocus model results. Panels (A) and (C): temporal dynamics of extents of local genomic divergence (truncated to the range indicated by the color bar) in single stochastic realizations of the model for weak selection (A) and for strong selection (C). The gray lines show the corresponding total extents of genomic divergence (the values are given on the *y*‐axis on the right). Panels (B) and (D): correlations of extents of divergence at pairs of loci as a function of their distance (measured in units of the recombination rate, *r*) averaged over 90 independent realizations for the parameters in (A) and (C), respectively. Correlations are color coded (see the color bar). Gray lines show the corresponding total extents of divergence averaged over 90 independent realizations. Other parameter values: selection parameter σ=4 (in A and B) or σ=2.5 (in C and D); population size, N=1000; mutation rate, $\mu =2\times 10^{-5}$; root mean square of mutation‐effect sizes, $\sigma_{\mu }=0.05$; migration rate, m = 0.1; recombination rate between a pair of adjacent loci, r=0.0005; number of adaptive loci, L=100. Note that the timescales in the upper panels differ from those in the bottom ones. (To interpret the references to color in this figure caption, the reader is referred to the web version of this article.)

In summary, earlier studies are partly contradictory. Indeed, Yeaman and Whitlock (2011) demonstrate late formation of clusters of divergence while Feder et al.’s (2012) theoretical arguments predict the opposite. Moreover, and in contrast to both Yeaman and Whitlock (2011) and Feder et al. (2012), Yeaman's (2013) theoretical analysis excludes any possibility for the emergence of clusters of differentiation during divergence‐with‐migration, unless specific recombination‐suppressor mechanisms are at work (factors that neither Yeaman and Whitlock 2011 nor Feder et al. 2012 included in their models). Critically, the existing theory relies on the assumption that any established local genomic divergence persists indefinitely, or increases due to the accumulation of new beneficial mutations. But this may not be the case in populations of finite size where genetic drift causes stochastic fluctuations of allele frequencies potentially leading to fixation of one (and the same) allele in diverging populations. Such a fixation event corresponds to loss of already established genomic divergence. This stochastic loss is of fundamental importance in all natural populations due to their finite sizes, and yet it is not known how loss influences patterns of genetic differentiation that arise during divergence‐with‐migration. Furthermore, because it is probably the case that in a majority of natural populations biological traits are controlled by a large number of loci (see, e.g., reviews by Phillips 2008, and by Wagner and Zhang 2011), to interpret empirical data, it is necessary to understand: What are the genomic signatures of the process of stochastic loss during divergence‐with‐migration when many loci underlie the phenotype under selection? Does stochastic loss contribute to the formation of clusters of differentiated loci and, if so, how strongly? Finally, does the effect of this process change as divergence is ongoing?

To answer these questions, we analyze a multilocus model of divergence‐with‐migration, similar to that used by Yeaman and Whitlock (2011). By contrast to Yeaman and Whitlock (2011) and Yeaman (2013), we find that small, tightly linked clusters of differentiated loci are necessary to initiate successful divergence under weak selection and high migration. Notably, these clusters form without invoking any specific mechanisms that reduce recombination. Furthermore, we show that clusters grow rapidly during the early stage of divergence, but shrink in size during the late stage. Under strong selection, by contrast, we find that clusters are not essential for divergence and that they instead form in the late stage of the process. Recall that increasing concentration in the late stage of the process has been reported by Yeaman and Whitlock (2011), but the formation and dynamics of clusters preceding this stage that we find under weak selection and strong migration has, to our knowledge, not been reported or explained elsewhere. To explain these results, we analyze a two‐locus model. We show that the balance between “stochastic loss” and “gain” of local genomic divergence in finite populations is a universal mechanism that governs the formation and temporal dynamics of clusters of differentiated loci. We stress that this is a *universal* mechanism because it is at work in all natural populations and unlike, for example, chromosomal rearrangements, it is not restricted to parts of the genome where specific recombination suppressors are active.

## Materials and Methods

### MULTILOCUS MODEL

We simulate a multilocus model of divergence between two diploid populations. The model is similar to that used in Yeaman and Whitlock (2011; see also Supporting Information S1). The two populations are assumed to occupy a pair of demes that are exposed to different environmental conditions, so that the phenotype is subject to opposing selection pressures in the two demes. We assume that in each deme (denoted by k=1,2) there is an optimal value $\theta^{\left(k\right)}$ for the phenotype. For simplicity, the two optima are assumed to be constant during time and symmetric around zero, so that $\theta^{\left(1\right)}=-\theta^{\left(2\right)}$.

We assume that the phenotype of an individual is determined by its diploid genotype at *L* loci arranged on a single chromosome (but we also analyze the model with loci spread across two chromosomes, see Supporting Information). In the model each allele is attributed an allele‐effect size by which it contributes additively to the phenotype of an individual. In other words, the phenotype $z_{i}$ of individual *i* equals the sum of allele‐effect sizes at the *L* loci. We assume that the fitness $w_{i}^{\left(k\right)}$ of individual *i* in population *k* (k=1,2) depends on the phenotype $z_{i}$ of this individual as:

(1) $$w_{i}^{\left(k\right)}=e^{-\frac{\;{\left(z_{i}-\theta^{\left(k\right)}\right)}^{2}}{2\;\sigma^{2}}}.$$

Here σ is a parameter that determines the width of the distribution of the surviving phenotypes (Sadedin et al. 2009). When σ is large, selection is weak and vice versa. The selection parameter σ is assumed to be constant during time and equal in the two populations. The fitness of an individual determines the contribution of this individual to the pool of offspring through soft fecundity selection. The soft‐selection assumption assures that the number of juveniles *N* surviving to maturity in a given deme is constant over time, and we assume that it is equal in the two populations. Generations are assumed to be discrete and nonoverlapping. The lifecycle of individuals is modelled as follows. Virgin adults migrate to the opposite deme at a per generation per individual rate *m*. Migration is followed by random mating locally within each population, recombination, and selection. Recombination is assumed to occur at a rate *r* between adjacent loci, per gamete, per generation. Finally, mutations accumulate at a rate μ per allele, individual, generation. Each mutation is given a mutation‐effect size by which it additively contributes to the effect size of the allele it lands on. Mutation‐effect sizes are drawn randomly from a Gaussian distribution with a standard deviation $\sigma_{\mu }$, and a mean zero. To check whether the results are robust against the model for mutation‐effect sizes, we also perform simulations in which mutation‐effect sizes are drawn from an exponential distribution mirrored around zero, so that the mean mutation‐effect size is zero. In these simulations the parameter of the exponential distribution is set to $\sqrt{2}/\sigma_{\mu }$ so that the variance of mutation‐effect sizes is equal to $\sigma_{\mu }^{2}$. Finally, we note that the analysis in Martin and Lenormand (2006) of empirical data on fitness effects of mutations in different environments (data taken from various species) suggests that predictions of a model with a Gaussian fitness function and Gaussian distributed mutation‐effect sizes are in good agreement with a majority of the empirical data tested.

### PARAMETER CHOICES

At the start of a simulation, all individuals at all loci are assumed to have alleles of effect size zero. We set arbitrarily $\theta^{\left(1\right)}=-\theta^{\left(2\right)}=2$ (Table 1). In the majority of simulations, the number of loci *L* is set to L=100, but we also test the model with L=2000 (Supporting Information). The parameter σ is chosen to account for weaker (σ=4) or stronger selection (σ=2.5). For further details on selection parameters, see Supporting Information S1. To assess how the patterns are influenced by the local population size *N*, we contrast results obtained with N=1000 and 200. The migration rate *m* is set to a high value (m=0.1) that allows us to capture the signatures of migration under the chosen values of other model parameters. The recombination rate *r* between a pair of adjacent loci is set to r=0.0005 or 0.001 so that the first and the last locus in the genomic region simulated (with L=100 loci) are at a recombination distance of about 0.05 or 0.1, respectively (but the distance is larger for L=2000). Note that r=0.0005 corresponds to about $5\;\times 10^{4}$ base pairs assuming that recombination rate between two nearby base pairs is 10^−8^. The mutation rate μ per generation, allele, locus, individual is chosen so that mutations that influence an individual's phenotype occur infrequently ($\mu =2\;\times \;10^{-5}$). Finally, the variance of mutation‐effect sizes $\sigma_{\mu }^{2}$ is set to a small value ($\sigma_{\mu }=0.05$) so that the square root of the total variance over all adaptive loci ($\sqrt{L}\sigma_{\mu }$) is smaller than the distance between the optimal trait values $\theta^{\left(1\right)}-\theta^{\left(2\right)}$. For the parameters set here and assuming that the whole genome region simulated acts as a single locus (total recombination rate is equal to zero), this means that it requires, on average, about four adaptive steps for the populations to reach their optimal traits (taking into account diploidy). Otherwise, if the distance between the optima is equal to or lower than $\sqrt{L}\sigma_{\mu }$, perfect adaptation in both populations can, by chance, be attained in a single adaptive step, which we consider to be an unlikely scenario in natural populations.

**Table 1:** Parameters of the model, and the values used in our computer simulations

Symbol	Explanation	Values
*N*	Population size per patch	$1000^{*},\;200$
*L*	Number of adaptive loci	$100^{*},\;2000$
*m*	Migration rate	0.1*
$\theta^{\left(k\right)}$	Optimal phenotype in population k=1,2	$\theta^{\left(1\right)}=2^{*},\;\theta^{\left(2\right)}=-2^{*}$
σ	Selection parameter	$4^{*},\;3.5,\;2.5^{*}$
*r*	Recombination rate	$0.0005^{*},\;0.001$
μ	Mutation rate	$2\;\times 10^{-5*},\;10^{-4}$
σ_μ_	Root mean square of mutation‐effect sizes	$0.05^{*},\;0.05/\sqrt{20}$

The symbol “*” indicates the parameter values used for the results shown in the main text. Results for other parameter values are shown in Supporting Information.

The model is simulated for a large number of generations (up to 10^5^) to allow the populations to come close to their local optima and stabilize. At intervals of 50 generations, we measure the extents of local and total genomic divergence as follows. The extent of local genomic divergence $D_{l}$ at locus *l* is estimated as twice the difference between the allele‐effect size of the most frequent alleles at this locus in the two populations. The factor 2 is used because the population is diploid. Our measure of local genomic divergence divided by 2 corresponds to the measure *d* used by Yeaman and Whitlock (2011). We approximate the extent of total genomic divergence *D* in a given generation by summing the extents of local genomic divergence at all loci in this generation. For our parameters, perfect adaptation in both populations corresponds to the total genomic divergence equal to the difference between the local optima $\theta^{\left(1\right)}-\theta^{\left(2\right)}=4$. As an alternative to the measure $D_{l}$ of the extent of local genomic divergence at locus *l*, one can use twice the average allele‐effect size at this locus (and the sum over all loci would correspond to the average total extent of divergence). We note that the two measures of divergence (one based on the most frequent alleles, and the other on the average allele‐effect sizes in the two populations) give rise to qualitatively the same patterns of divergence (see below). However, because the measure *D* is directly comparable to the measure *d* used by Yeaman and Whitlock (2011), we present most of our results in terms of this measure. Divergence patterns presented similarly to Yeaman and Whitlock (2011, see their Fig. 3) allow one to inspect visually each individual realization of the model and to evaluate roughly whether clusters of divergence are formed and, if yes, what is their typical size. Here, however, we complement such a visual inspection by measuring correlations of local extents of genomic divergence at pairs of loci as a function of their recombination distance. This allows us to capture the extent of similarity of differentiation at pairs of loci at various recombination distances in a given generation. Note that when a cluster forms, the extents of divergence at loci within the cluster are expected to be more correlated than the extents at loci outside of the cluster. Therefore, if a cluster is formed, its size (i.e., the recombination distance it spans) is expected to be captured by the recombination distance at which the correlation function decays to values close to zero. For each parameter set, we run 90 independent simulations (unless otherwise noted) to evaluate the effect of stochastic fluctuations on the extents of local and total divergence.

**Figure 2:**
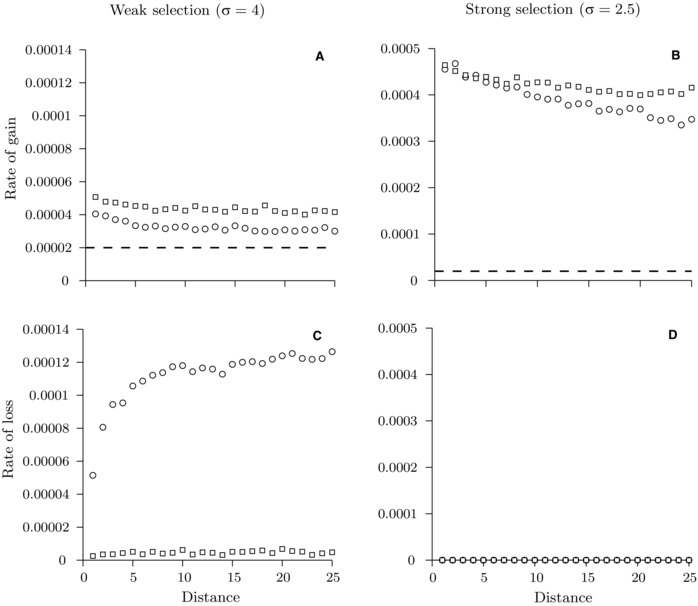
Rate of gain and rate of loss in the two‐locus gain–loss model with one weakly diverged locus ($D_{w}=0.2$) and a more strongly diverged one ($D_{s}=0.4$). Shown are the rates as a function of distance between the loci (measured in units of recombination rate, *r*). Panels (A) and (B): rate of gain at the weakly diverged locus (circles) and at the more strongly diverged locus (squares) for weak selection (A), and for strong selection (B). Dashed lines indicate the mutation rate μ (this rate corresponds to the rate at which a neutral mutation lands and fixates at a neutral locus in a diploid population of size *N*). Panels (C) and (D): corresponding rates of loss for the parameters in (A) and (B), respectively. Circles and squares overlap in (D). Other parameter values: selection parameter σ=4 in (A) and (C) or σ=2.5 in (B) and (D); population size in each deme, N=1000; migration rate, m=0.1; mutation‐effect size, ε=0.05; recombination rate, r=0.0005; mutation rate, $\mu =2\times 10^{-5}$. Number of simulations used are as follows: 2 × 10^6^ in (A), 5 × 10^5^ in (B), 10^3^ in (C), and 200 in (D).

### TWO‐LOCUS MODEL

To understand the mechanisms at work in the multilocus model presented above, we analyze a two‐locus model. In particular, we use two versions of a two‐locus model. One is an *establishment model* (similar to the models used by Feder et al. 2012, and by Yeaman 2013), and the other is a (novel) *gain–loss model*. These two are briefly explained next (but see also Supporting Information S2 and S3).

As noted in the introduction, earlier theory of divergence‐with‐migration focuses on evaluating the importance of the establishment bias for the evolution of genetic architectures during divergence‐with‐migration (Yeaman and Whitlock 2011; Feder et al. 2012; Yeaman 2013). Recall that the establishment bias here means that the probability of establishment of a new mutation is larger for the mutation landing closer to an already diverged locus than further away. Although the establishment bias can be significant when very few loci underlie a selected trait (Yeaman and Whitlock 2011; Feder et al. 2012), this is not true when many loci underlie the trait (Yeaman 2013). To check whether this finding of Yeaman (2013) holds true for the parameter values used in this study (see Section “Parameter Choices”), we employ the establishment model analyzing whether there is a range of recombination distances around an already diverged locus such that a successful establishment of a new mutation is more likely within this range than outside of it (see below). In this study, *a successful establishment* of a mutation means that the mutant allele is most common (frequency >50%) in the deme where it is advantageous (cf. Yeaman and Otto 2011). In the establishment model, one locus is differentiated at the outset, the other is not (Supporting Information S2). We analyze the establishment probability of new mutations at the undifferentiated locus, varying its recombination distance from the differentiated one. Using these results, we compare the probability that a new mutation lands and establishes within a genomic region of a given size around the diverged locus to that outside of this region (as suggested earlier by Yeaman 2013). When the mutation rate per locus is equal for all loci (as we assume here and in accordance with Yeaman 2013), the ratio between the two probabilities is independent of the mutation rate, and it is equal to the integral of the establishment probability over recombination distances within a region relative to the integral of the establishment probability over recombination distances outside of this region (but within the total genomic region considered). If this ratio is greater than unity, we can immediately deduce that a cluster of divergence is likely to be formed. Otherwise, a new mutation may establish at any recombination distance from the differentiated locus. By contrast to Yeaman (2013), we argue, however, that in this case we cannot draw a final conclusion about cluster formation because, once established, any local genomic divergence is subject to two competing processes. One process is stochastic loss that occurs due to random genetic drift in populations of finite size, resulting in fixation of a single allele at a given locus in both populations. The other process is the gain of additional local genomic divergence that occurs due to the influx of new mutations followed by their successful establishment. The ratio of the rates at which local stochastic loss and gain operate (hereafter referred to as *balance*) determines whether divergence established in a genomic region at a given divergence stage will make a lasting contribution to overall differentiation. The balance between stochastic loss and gain at a given locus depends on the distance of this locus from other diverged loci in the genome, as well as the strength of local and total genomic divergence. To understand this dependence, we use the two‐locus gain–loss model.

In this model, both loci are assumed to have established divergence. One locus is assumed to be weakly differentiated with the extent of divergence *D*_w_ corresponding to the establishment of one mutation beneficial in the first population, and one mutation beneficial in the second one. We set the allele‐effect sizes at this locus to $Y_{w}=\sigma_{\mu }$, and $-Y_{w}$, so that $D_{w}=4\sigma_{\mu }$ (for further details on this choice, see Supporting Information S3). The second locus is assumed to have stronger divergence *D*_s_ ($>D_{w}$) with allele‐effect sizes *Y*_s_ ($>Y_{w}$), and $-Y_{s}$ (and so $D_{s}=4Y_{s}$). We vary the value of *Y*_s_ in the simulations to mimic different stages of divergence (see Supporting Information S4 for details on the initial conditions in these simulations). Using this model, we estimate the rate of local gain (product of the rate at which a mutation lands at a locus and the rate at which this mutation establishes successfully conditional on it landing at the locus), and the rate of local loss at the two differentiated loci. When the rate of loss at a locus is larger than the rate of gain at this locus in a given stage of divergence, this locus is unlikely to make a sustained contribution to overall divergence. Otherwise, the opposite is true. In what follows, we explain our method for estimating the rates of gain and loss using this model.

In the limit of rare mutations (2μN≪1) and when the two loci are at a recombination distance $r_{j}=jr$ (j=1,...,L), the rate of local gain $\lambda_{G,w}\left(r_{j}\right)$ at the weakly diverged locus is equal to the product of the probability that a locally beneficial mutation lands at this locus (2μN), and the probability $p_{G,w}\left(r_{j}\right)$ that it establishes at this locus (conditional on the mutation landing at the locus):

(2) $$\lambda_{G,w(r_{j})}=2\mu Np_{G,w}\left(r_{j}\right).$$

Substituting subscripts *w* in equation (2) by *s*, we obtain the corresponding expression for the rate of local gain at the more strongly diverged locus. In the limit of $\lambda_{G,w}\left(r_{j}\right)\ll 1$, the time to local gain at the weakly diverged locus is approximately exponentially distributed with mean $\lambda_{G,w}^{-1}\left(r_{j}\right)$ (and similarly for the more strongly diverged locus).

We estimate the rates of gain at the two loci using two separate sets of simulations. In one set, we assume that a mutation of a fixed mutation‐effect size, $\varepsilon =\sigma_{\mu }$ (as in the establishment model, see Supporting Information S2), lands at the weakly diverged locus immediately after the initialization of the system (the initial condition is explained in detail in Supporting Information S4). In the other set of simulations, we assume that the mutation lands on the more strongly diverged locus (all other settings are the same as in the former set of simulations). Further mutations are thereafter neglected. We further assume that in the former case the mutation lands in the first population (where it is beneficial) on an allele of effect size *Y*_w_ (and similarly on *Y*_s_ in the latter case). For these settings, the mutant allele is advantageous over both alleles at the locus prior to the mutation, and hence it will promote the local extent of divergence upon a successful establishment. We use a similar method to that explained in the establishment model (see above) to estimate the establishment probabilities $p_{G,w}\left(r_{j}\right)$, and $p_{G,s}\left(r_{j}\right)\;$at the weakly and at the more strongly diverged locus, respectively. Finally, we use equation (2) to estimate the rates of gain at the two loci.

In addition to the rates of gain, we estimate the rates of local loss at the two loci starting from the same initial condition as in the simulations described above, but now neglecting mutations. Each simulation is run under drift, selection, migration, and recombination until one or the other locus experiences loss of divergence or until a predetermined maximum time (*T*_m_) expires. As explained above, *loss of divergence* means that a locus becomes monomorphic due to fixation of one allele in both populations. Using simulations, we first estimate the probabilities $p_{L,s}\left(r_{j}|T_{m}\right)$ and $p_{L,w}\left(r_{j}|T_{m}\right)$ that the first loss event occurs at the more strongly or at the weakly diverged locus, respectively, conditional on it occurring before *T*_m_. Here, $r_{j}$ denotes the recombination distance between the two loci (see above). Second, we estimate the average time $\left\langle t_{L}\left(r_{j}|T_{m}\right)\right\rangle$ until the first loss event based on simulations in which a loss has occurred by the maximum time *T*_m_. Using these data, we estimate the rates of loss $\lambda_{L,s}\left(r_{j}\right)$ and $\lambda_{L,w}\left(r_{j}\right)$ at the more strongly and at the weakly diverged locus, respectively, based on the following considerations. In the limit of $\lambda_{L,s}\left(r_{j}\right)\ll 1$, the time to loss at the more strongly differentiated locus is approximately exponentially distributed with mean $\lambda_{L,s}^{-1}\left(r_{j}\right)$ (and similarly for the weakly diverged locus). Furthermore, in the limit of $\lambda_{L,s}\left(r_{j}\right)\ll 1$, $\lambda_{L,w}\left(r_{j}\right)\ll 1$, the time until the first loss event (either at the first or at the second locus) is approximately exponentially distributed with mean ${\left(\lambda_{L,s}\left(r_{j}\right)+\lambda_{L,w}\left(r_{j}\right)\right)}^{-1}$. Therefore, the probability $p_{L}\left(r_{j}|T_{m}\right)=p_{L,s}\left(r_{j}|T_{m}\right)+p_{L,w}\left(r_{j}|T_{m}\right)$ that the first loss event occurs either at the more strongly or at the weakly diverged locus by the time *T*_m_ is given by:

(3) $$p_{L}\left(r_{j}|T_{m}\right)=1-e^{-\left(\lambda_{L,s}\left(r_{j}\right)+\lambda_{L,w}\left(r_{j}\right)\right)T_{m}}.$$

Finally, we find that the average time $\left\langle t_{L}\left(r_{j}|T_{m}\right)\right\rangle$ to the first loss event, conditional on the loss occurring by the time *T*_m_, can be expressed in terms of $\lambda_{L,s}\left(r_{j}\right)$, $\lambda_{L,w}\left(r_{j}\right)$, $p_{L}\left(r_{j}|T_{m}\right)$, and *T*_m_ as follows:

(4) $$\left\langle t_{L}\left(r_{j}|T_{m}\right)\right\rangle =\frac{1}{\lambda_{L,s}\left(r_{j}\right)+\lambda_{L,w}\left(r_{j}\right)}-T_{m}\frac{1-p_{L}\left(r_{j}|T_{m}\right)}{p_{L}\left(r_{j}|T_{m}\right)}.$$

Because

(5) $$\frac{p_{L,s}\left(r_{j}|T_{m}\right)}{p_{L,w}\left(r_{j}|T_{m}\right)}=\frac{\lambda_{L,s}\left(r_{j}\right)}{\lambda_{L,w}\left(r_{j}\right)\;},$$

we obtain

(6) $$\lambda_{L,w}\left(r_{j}\right)=\frac{p_{L,w}\left(r_{j}|T_{m}\right)}{p_{L}\left(r_{j}|T_{m}\right)}\;{\left(\langle t_{L}\left(r_{j}|T_{m}\right)\rangle +T_{m}\frac{1-p_{L}\left(r_{j}|T_{m}\right)}{p_{L}\left(r_{j}|T_{m}\right)}\right)}^{-1}.$$

We use equation (6) to estimate the rate of loss $\lambda_{L,w}\left(r_{j}\right)$ given the probabilities $p_{L,s}\left(r_{j}|T_{m}\right)$ and $p_{L,w}\left(r_{j}|T_{m}\right)$, and the average time $\left\langle t_{L}\left(r_{j}|T_{m}\right)\right\rangle$ that we obtain using simulations. The rate of local loss at the more strongly diverged locus is obtained by combining equations (5) and (6).

Note that $\left\langle t_{L}\left(r_{j}|T_{m}\right)\right\rangle$ is not defined if no loss occurs by the maximum time *T*_m_ set in the simulations. To avoid such cases, *T*_m_ has to be long enough ($T_{m}\gg {\left(\lambda_{L,s}\left(r_{j}\right)+\lambda_{L,w}\left(r_{j}\right)\right)}^{-1}$) to assure that loss occurs by this time with a high enough probability. Because we do not know the rates $\lambda_{L,s}\left(r_{j}\right)$ and $\lambda_{L,w}\left(r_{j}\right)$ in advance, *T*_m_ has to be chosen. Here, we set it to a large value $T_{m}=10^{5}$, because this allows us to compare the timescales of local loss and gain for other parameter values used in this study. Indeed, when the mutation rate is $\mu =2\;\times 10^{-5}$ (as in Fig. 1), this value of *T*_m_ corresponds to twice the average waiting time until a mutation establishes successfully at a neutral locus in an isolated population. Hence *T*_m_ is larger than the average time to local gain at any locus under selection (inverse of the rate of gain, see above). Importantly, in situations when no loss occurs by this time, we immediately deduce that the rate of local loss is much smaller than the sum of rates of gain at the weakly and at the more strongly diverged locus according to eq. (3).

## Results

Under weak selection (Fig. 1A, B), we find an initial phase of roughly homogeneous divergence over the adaptive loci with the pairwise correlation of divergence being independent of the recombination distance between loci (Fig. 1B). In this phase, divergence at any one locus is highly transient and the total extent of divergence is very low. After a waiting time of about 10,000 generations (in this particular realization, but see other examples in Fig. S1), groups of closely linked loci establish divergence. This initiates rapid formation of a cluster of divergence that extends in size and immediately promotes the advance of phenotypic adaptation. At about half way toward perfect adaptation (D≈2), the cluster of diverged loci attains a maximum size (Figs. 1A and S1), with an average of around 15 loci (Fig. 1B). Thereafter, the cluster shrinks in size, but most of the cluster still remains after 10^5^ generations.

Under strong selection, the initial phase is also characterized by roughly uniformly distributed divergence (Fig. 1C, D). The buildup of divergence is, as expected, much faster under strong selection, and the formation of a cluster is not necessary to initiate population divergence. Even so, when approximately perfect adaptation is attained under strong selection (D≥4), divergence starts to concentrate, resulting in formation of a cluster of divergence. Note that, comparing to weak selection, a cluster under strong selection starts forming in a much later stage of divergence in terms of the value of *D*, but sooner in terms of the number of generations after the start of divergence (2000 rather than 10, 000 generations in the particular realizations shown in Fig. 1A and C, respectively). Note also that the divergence patterns obtained using exponentially distributed mutation‐effect sizes, with otherwise the same settings as those in Figure 1, do not qualitatively differ from the patterns shown in Figure 1 (see Fig. S9). The same is true if local extents of genomic divergence are measured using the average allele‐effect size instead of the measure used in Figure 1 (see Fig. S10).

To investigate further the differences in divergence patterns obtained for weak and strong selection (Fig. 1A and C, respectively), we estimate the establishment probabilities of a new mutation as a function of the recombination distance from an already diverged locus (Supporting Information Fig. S2). Next, we compare the probability that a mutation lands and establishes outside of a genomic region around the diverged locus to the probability that it lands and establishes inside of this region (Supporting Information Fig. S3). For a small genomic region surrounding the diverged locus, we find that the probability of landing and establishment outside of the region is much larger than the corresponding probability inside of the region. Furthermore, even when the region accounts for 50% of the whole genomic region simulated (100 loci), the corresponding probability outside is only slightly less than the probability inside of the region. These findings (consistent with Yeaman 2013) are true both for weak and strong selection, suggesting that the establishment bias is too weak to cause the formation of clusters, especially the tightly linked ones observed under weak selection in our multilocus simulations. Consequently, we need an additional mechanism to explain the emergence of a cluster under weak selection.

Our gain–loss model helps to understand the progress of cluster formation. In this two‐locus model, both loci already have some divergence established, but one has diverged more strongly ($D_{s}\geq 0.4$) than the other ($D_{w}=0.2$). First, analyzing the rate of gain at the two loci in an initial stage of the divergence process (D=0.6, 15% of the value corresponding to perfect divergence), we find that the rate is marginally larger at the more strongly diverged locus (Fig. 2A, B). For both loci, the rate of gain of new genetic differentiation is higher when the two loci are at a smaller recombination distance but this distance dependence is weak for either weak or strong selection (Fig. 2A, B). Recall that the rate of gain is the product of the mutation rate and the establishment probability of a new mutation conditional on it landing locally in the genome. Therefore, the rate of gain depends weakly on the recombination distance between the loci due to a weak bias in the establishment probability discussed above.

Second, we analyze the risk of loss of divergence by stochastic processes that may eliminate variation in either of the two loci. Under weak selection, we show that the rate of loss of divergence at the more diverged locus is small and depends only weakly on the recombination distance between the two loci (Fig. 2C, squares). However, at the less diverged locus the rate of local loss increases rapidly with recombination distance from the other locus (Fig. 2C, circles). Comparing the rate of gain and loss at the weakly diverged locus, we find a critical recombination distance from the more strongly diverged locus above which loss is on average faster than gain. Divergence established at a less diverged locus above this critical distance is unlikely to make a lasting contribution to the overall divergence. In fact, in this initial stage of divergence, the rate of loss is marginally larger than the rate of gain already at one locus distance from the more differentiated locus (where distance is scaled by the recombination rate between a pair of adjacent loci). As a consequence, in most cases a weakly diverged locus adjacent to a more strongly diverged one fails to contribute to further divergence. However, occasionally, as a matter of chance and after a shorter or longer waiting time, divergence is gained at the weakly diverged locus. By contrast, under strong selection no loss of divergence occurred during 200 simulations at either of the two loci, and so in this case the rate of local loss is much smaller than the rate of local gain for all between‐locus recombination distances that we analyzed (Fig. 2D).

**Figure 3:**
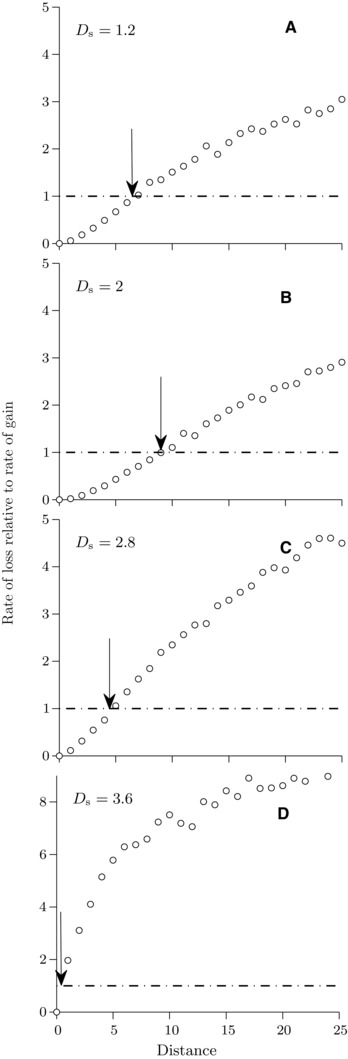
Rate of loss relative to rate of gain in the two‐locus gain‐loss model for weak selection at different stages of divergence (different *D*_s_) as a function of distance between the loci (measured in units of the recombination rate, *r*). Dash‐dotted lines correspond to a ratio of unity. The arrow in each panel depicts an approximate location of the critical distance above which the rate of loss is larger than the rate of gain for the corresponding value of *D*_s_. Selection parameter: σ=4. Number of simulations used: 2 × 10^6^ for the rate of gain, 1000 for the rate of loss. Other parameter values are the same as in Figure 2.

**Figure 4:**
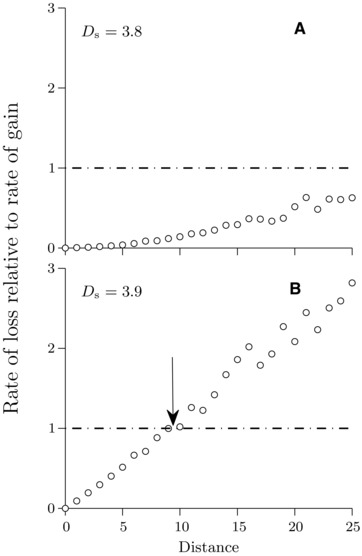
Same as in Figure 3, but for strong selection (σ=2.5). Both panels are for late stages of divergence ($D_{s}=3.8$ in [A], and $D_{s}=3.9$ in [B]). Number of simulations used for the rate of gain: 5 × 10^5^. Number of simulations used for the rate of loss: 200 in (A) and 1000 in (B). Other parameter values are the same as in Figure 2.

After the initial stage, the divergence rapidly continues to increase both under weak and strong selection (Fig. 1). This alters the balance between rates of loss and gain. From the two‐locus model, we find that under weak selection, the critical recombination distance between the two loci below which there is a net gain of new divergence, initially increases leading to an overall increase in divergence and cluster size (compare positions of arrows in Fig. 3A–D). Half way toward perfect local adaptation ($D_{s}\approx 2$), the critical distance starts to decrease. Consequently, under weak selection and as *D*_s_ increases up to $D_{s}\approx 2$, the cluster grows reaching the size of about 20 loci (two times the maximum critical scaled distance of 10 loci in Fig. 3B). This largely corroborates the finding of the multilocus model, where the maximum cluster size attained during divergence is about 15 loci on average. Thereafter, the two‐locus model predicts that the cluster will shrink in size and this is also observed in the multilocus model (Fig. 1A). Under strong selection, by contrast, there is a continued net gain of divergence until populations approach perfect adaptation (D≈4, Fig. 4A). At this stage, both populations have at their disposal gene variants that combined together give rise to locally perfectly adapted individuals. However, stochastic loss of divergence becomes increasingly important for alleles of small effect that are loosely linked to loci with stronger divergence (Fig. 4B). Adaptation is maintained by gain at closely linked loci, leading to increasing clustering. The key features of cluster formation early in divergence and decrease in cluster size later in divergence are retained for higher recombination rate, intermediate selection, lower population size, and larger genomic region (Supporting Information Figs. S4–S8).

## Discussion

Reproductive isolation between populations is most efficient when many small barriers to gene flow are formed throughout the genome (Barton 1983; Coyne and Orr 2004). Otherwise, linkage to barrier loci may be insufficient to prevent gene flow over a large part of the genome (Barton and Bengtsson 1986). Thus, the genomic distribution and effect sizes of loci underlying local adaptation are critical to understanding the origin of reproductive isolation in models of ecological speciation with gene flow (Nosil 2012; Seehausen et al. 2014).

Models of divergence are attractive in the sense that they suggest different mechanisms by which the barrier effects of single adaptively divergent loci may be enhanced so that the total barrier increases and the genomic region affected broadens (Via 2009; Feder et al. 2012). However, recent simulation studies suggest that local barrier effects are enhanced only extremely late in the process (Yeaman and Whitlock 2011), or that they are unlikely to be of any biological relevance unless interacting with chromosomal inversions and other genomically localized mechanisms that reduce recombination (Feder et al. 2013; Yeaman 2013). In contrast to these conclusions, we here show that a specific mechanism that suppresses recombination is not necessary for clusters of differentiation to form. Moreover, we show that under weak selection and strong migration, the emergence of a concentrated genetic architecture is indispensable for phenotypic divergence to evolve. These findings are, to our knowledge, new and contribute to explaining observed empirical patterns, as discussed below.

The reason we detect clusters of differentiation despite the fact that the establishment bias (referred to as “divergence hitchhiking” by some authors; Feder et al. 2012; Yeaman 2013) is too weak to support clustering is because a mechanism beyond the establishment bias is at work. In contrast to the establishment probability, the rate of loss of differentiation at a weakly diverged locus depends strongly on the recombination distance to a locus of stronger effect, and so the balance between loss and gain of small extents of local differentiation also depends strongly on the recombination distance. This balance between loss and gain is the key mechanism underlying the formation of clusters of divergence.

Our results show that when new locally beneficial mutations are under weak selection and migration between the diverging populations is frequent, tightly linked clusters of differentiated loci are a prerequisite for initialization of successful phenotypic divergence. The initialization occurs after a waiting time that is, on average, longer for parameter settings giving rise to smaller clusters, that is, weaker selection, smaller variance of mutation‐effect sizes, smaller population sizes, higher migration rate. However, when selection for locally beneficial mutations is sufficiently strong, we find rapid phenotypic divergence that precedes cluster formation.

Our two‐locus analysis of the interplay between loss and gain of local genomic divergence is highly consistent with the results of the multilocus modeling. A key idea is that any locus that has established divergence may either risk a stochastic loss of divergence (similar to the idea of “transient divergence” Yeaman 2015) or gain from additional beneficial mutations. Consequently, a diverged locus is, at a given stage, unlikely to make a lasting contribution to the overall divergence if the rate of local loss is larger than the rate of local gain. There is a critical recombination distance from the focal locus above which local loss is faster than local gain. This distance depends on the selection strength and it varies over the time frame of the divergence process. Under weak selection, the critical distance increases early in the divergence process but, about half‐way to perfect adaptation, it starts to decrease. The latter effect arises because, after about half‐way to perfect adaptation for the parameter values we tested, a weakly differentiated locus at a given recombination distance from other differentiated loci contributes proportionately by a very small amount to the overall extent of divergence and to the reduction of gene flow between the populations. This contribution becomes smaller as the total extent of divergence increases beyond a point corresponding to about half‐way to perfect adaptation. Consequently, as divergence progresses above this point, the rate of gain of new differentiation at a given weakly differentiated locus decreases, and the rate of loss increases. Therefore, the ratio between the rate of local loss and the rate of local gain increases, resulting in shrinking of a cluster over time. For a similar reason genetic architectures concentrate in late stages of the divergence process also under stronger selection (or weaker migration) but this occurs later in the divergence process. In particular, under strong selection considered here (see also Yeaman and Whitlock 2011), diverging populations attain almost perfect adaptation before clustering of the genetic architecture starts. The dynamics of clusters obtained under our multilocus simulations is, therefore, consistent with the main predictions of the two‐locus gain–loss model in different stages of divergence. Notably, because our analysis contrasts the effects of loss and gain locally in the genome, the consequences of the balance between these two effects for the size of a cluster (i.e., the recombination distance it spans) in multilocus models of divergence is independent of the number of selected loci, provided that this number is large.

We find that the cluster size emerging in our model is well characterized by the correlation function describing the similarity in extents of divergence in pairs of loci in relation to their recombination distance. When clusters are formed, the correlation decreases with increasing recombination distance between the loci, reaching approximately zero at the cluster margin. This measure is closely related to measures of linkage disequilibrium (McVean 2002; Eriksson and Mehlig 2004; Schaper et al. 2012) that are frequently used in empirical studies (Smadja and Butlin 2011; Martin et al. 2013).

Apart from the findings discussed above, we also find that when multiple tightly linked clusters emerge during divergence (see an example in Fig. S1A), the clusters compete with each other for gaining new differentiation (or against losing the differentiation they have established). The dynamics of such a competition can be investigated by a gain–loss model similar to that analyzed here, but with more than two loci included and focusing on gain and loss of differentiation at individual clusters, each of which contains multiple loci.

Some modeling studies have earlier considered the loss of divergence. Using a single‐locus model, Yeaman and Otto (2011) found that less diverged loci have a smaller persistence time than more diverged ones. A single‐locus analysis is, however, insufficient to explain clustering, because it is not only the extent of local divergence that matters, but also the extent of divergence at other diverged loci and their linkage. In addition, it is not the persistence time *per se* that matters but, as shown here, a balance between loss and gain processes, which operates differently in different stages of divergence. In a recent study, Aeschbacher and Bürger (2014) analyzed a two‐locus continent‐island model of divergence, deriving an approximation for the mean extinction time of a mutation at some recombination distance from a diverged locus. Comparing the mean extinction time at a linked locus with that at an unlinked one, they showed that the mean extinction time is shorter when linkage is looser. However, this comparison may not be relevant for the patterns of divergence because, as we show, the balance between local loss and gain shifts over the timescale of the process.

Due to our upper limit of 10^5^ generations, we do not capture the final fate of the clusters. Yeaman and Whitlock (2011) suggested that a pair of populations undergoing divergence‐with‐migration will eventually differ at a single locus, and our results seem to corroborate this conclusion. We note, however, that other factors, such as the evolution of habitat choice or assortative mating, may reinforce isolation (Thibert‐Plante and Gavrilets 2013). These processes are likely to prevent clustering in late stages of divergence by reducing gene flow between populations and introducing additional mechanisms at work (Cruickshank and Hahn 2014).

Empirical studies report either little evidence of genomic clustering (Soria‐Carrasco et al. 2014), or strong evidence for generally small clusters (Jones et al. 2012), or for two orders of magnitude larger clusters (Ellegren et al. 2012). This large variation in cluster size may hint that different mechanisms are involved (Seehausen et al. 2014), including those that reduce recombination (Yeaman 2013). Theory suggests that inversions might be more important than other recombination suppressors because they work specifically in heterozygotes, rather than generally suppressing recombination (Otto and Lenormand 2002). However, there is evidence for fine‐scale variation in recombination rates that is also likely to contribute to heterogeneous patterns (Burri et al. 2015).

In general, the extent of migration between the diverging populations is an important factor shaping the genetic architectures evolving during divergence (Feder et al. 2013, Seehausen et al. 2014). For example, in a recent study by Marques et al. (2016), it has been shown that genetic differentiation between sympatric races of three‐spine sticklebacks is concentrated in the genome, occurring over few very short genomic regions on only two chromosomes (see their Fig. 3C). By contrast, many more differentiated genes and chromosomes are detected between essentially allopatric races of this species, suggesting a roughly uniformly distributed differentiation (see Fig. 3D in Marques et al. 2016). These results seem consistent with the predictions of our model comparing high and small migration rate between the diverging populations. However, Marques et al. (2016) also suggest that the diverging populations they analyzed probably had some amount of standing genetic variation at the time they were introduced to the sites examined. The role of standing genetic variation, however, is not examined by our current model, and we find this to be an important future avenue.

In summary, of the different mechanisms potentially contributing to the formation of local barriers to gene flow, the balance between the processes of local loss and gain that we proposed here is, to our knowledge, the only universal mechanism that promotes concentrated genetic architecture under strong gene flow, without suppressing recombination. We show that the number of loci in a cluster is smaller under weaker selection, smaller mutation‐effect sizes, smaller population size, and stronger recombination. All of these parameters are likely to vary among species, and among populations within species. Furthermore, our model predicts systematic changes in cluster size during divergence. Thus, the balance between loss and gain of local genomic divergence potentially explains much of the observed variation in genomic architectures emerging during divergence‐with‐migration and leads to testable predictions about the causes of this variation.

## Supporting information


**Figure S1**. Same as in Figure 1A in the main text, but here patterns from two different realizations are shown.
**Figure S2**. Results of the two‐locus establishment model.
**Figure S3**. Establishment bias in the two‐locus establishment model.
**Figure S4**. Patterns of divergence under the parameter values similar to those in Figure 1A and B in the main text, but here the recombination distance between adjacent loci is two times larger (*r* = 0.001).
**Figure S5**. Effect of drift.
**Figure S6**. Same as in Figure 3 in the main text but for the selection parameter σ = 3.5 (corresponding to that used in Fig. S5).
**Figure S7**. Patterns of divergence under the parameter values similar to those in Figure 1C in the main text, but with 20 times more adaptive loci (*L* = 2000), and 20 times smaller variance $\sigma_{\mu }^{2}$ of mutation‐effect sizes.
**Figure S8**. Same as in Figure 3 in the main text, but for the parameters corresponding to those in Figure S7.
**Figure S9**. Patterns of divergence for the parameter values corresponding to those in Figure 1 in the main text, but here mutation‐effect sizes are drawn from an exponential distribution mirrored around zero (i.e., positive and negative effects are assumed to be equally likely).
**Figure S10**. A comparison between patterns of divergence in a single stochastic realization of the model, but shown using two different measures for the extent of divergence at locus *l*, that is, in (A) we use the measure *D_l_* introduced in the main text (the total extent of divergence is equal to $\sum_{l=1}^{L}D_{l}$), and in (B) we use instead twice the difference between average allele‐effect sizes at locus *l* in the two populations (the average extent of divergence is equal to the sum of average allele‐effect sizes at all *L* loci simulated).
