# Supplementary material for: A universal mechanism generating clusters of differentiated loci during divergence‐with‐migration
Source: Evolution. 2016 Jun 1;70(7):1609–21. doi: 10.1111/evo.12957 (PMC5089645; doi:10.1111/evo.12957)
Supplement: Supplementary file 1 — Figure S1. Same as in Figure 1A in the main text, but here patterns from two different realizations are shown. Figure S2. Results of the two‐locus establishment model. Figure S3. Establishment bias in the two‐locus establishment model. Figure S4. Patterns of divergence under the parameter values similar to those in Figure 1A and B in the main text, but here the recombination distance between adjacent loci is two times larger (r = 0.001). Figure S5. Effect of drift. Figure S6. Same as in Figure 3 in the main text but for the selection parameter σ = 3.5 (corresponding to that used in Fig. S5). Figure S7. Patterns of divergence under the parameter values similar to those in Figure 1C in the main text, but with 20 times more adaptive loci (L = 2000), and 20 times smaller variance σμ2 of mutation‐effect sizes. Figure S8. Same as in Figure 3 in the main text, but for the parameters corresponding to those in Figure S7. Figure S9. Patterns of divergence for the parameter values corresponding to those in Figure 1 in the main text, but here mutation‐effect sizes are drawn from an exponential distribution mirrored around zero (i.e., positive and negative effects are assumed to be equally likely). Figure S10. A comparison between patterns of divergence in a single stochastic realization of the model, but shown using two different measures for the extent of divergence at locus l, that is, in (A) we use the measure Dl introduced in the main text (the total extent of divergence is equal to ∑l=1LDl), and in (B) we use instead twice the difference between average allele‐effect sizes at locus l in the two populations (the average extent of divergence is equal to the sum of average allele‐effect sizes at all L loci simulated). [file EVO-70-1609-s001.pdf]

# Supplementary information for "A universal mechanism generating clusters of differentiated loci during divergence-with-migration"


Marina Rafajlović[1,2,*], Anna Emanuelsson[1], Kerstin Johannesson[3,2], Roger K. Butlin[4,2], and Bernhard Mehlig[1,2]

[1]*Department of Physics, University of Gothenburg, SE-412 96 Gothenburg, Sweden*

[2]*The Linnaeus Centre for Marine Evolutionary Biology, University of Gothenburg, SE-405 30 Gothenburg, Sweden*

[3]*Department of Marine Sciences, Tjärnö, University of Gothenburg, SE-452 96 Strömstad, Sweden*

[4]*Department of Animal and Plant Sciences, University of Sheffield, Sheffield S10 2TN, UK*

[*]*Corresponding author,* marina.rafajlovic@physics.gu.se

## S1. DETAILS ABOUT SELECTION PARAMETERS USED IN THE MAIN RESULTS

In the main text, patterns of divergence for the model introduced in **Materials and Methods** are shown for two values of the selection parameter $\sigma$, that is, for $\sigma = 4$, and $\sigma = 2.5$. Selection is weaker in the former than in the latter case. In either case, the optimal trait values $\theta^{(1)}$ and $\theta^{(2)}$ in the two demes are set to $\theta^{(1)} = -\theta^{(2)} = 2$. Therefore, for $\sigma = 4$ we find that a perfectly adapted individual in one deme experiences a fitness disadvantage of about $0.39$ in comparison to perfectly adapted individuals in the other deme, and the fitness disadvantage of the first generation hybrids between perfectly adapted individuals in the two demes is about $0.12$ (also in relation to locally perfectly adapted individuals). Note that in earlier stages of divergence, the fitness disadvantage of the first generation hybrids in comparison to locally favourable individuals (that have not reached their optima) in either deme is smaller than $0.12$. In this respect, the extent of divergent selection in all stages of divergence under $\sigma = 4$ in our model corresponds to weak levels of selection under the model of Feder et al. (2012) ($s_{\rm o} < 0.12$ in their model). Under the stronger selection ($\sigma = 2.5$) in our model, the corresponding fitness disadvantage of individuals that are perfectly adapted for the opposite deme, and of the first generation hybrids are about $0.72$, and $0.27$, respectively.

We also note that when selection is weak, that is, when $\sigma^2$ is large in comparison to the distance between the optima $\theta^{(1)} - \theta^{(2)}$, our fitness function (Eq. (1) in the main text) reduces to that used by Yeaman and Whitlock (2011), with $\gamma = 2$, $\theta^{(1)} = -\theta^{(2)} = \theta$, and $\Phi = 2\theta^2/\sigma^2$ in their model. In particular, the selection strength corresponding to setting $\sigma = 2.5$ in our model is similar to that used by Yeaman and Whitlock (2011) for their parameter $\Phi = 0.75$ (see above).

Finally, the standard deviation $\sigma_\mu$ of the Gaussian distribution from which mutation-effect sizes are drawn is set to $\sigma_\mu = 0.05$ in the main text. With this choice, we find that in the first population (with the positive optimal phenotype) the selective advantage of a heterozygote with allele-effect sizes $0|0.05$ (the latter corresponding roughly to one mutation of effect-size $0.05$ landing on an allele of effect size $0$) over the homozygote with allele-effect sizes $0|0$ is about $0.006$ for the weaker selection tested ($\sigma = 4$), whereas for the stronger selection ($\sigma = 2.5$) it is about $0.016$ (which is approximately three times larger than under the weaker selection).

## S2. TWO-LOCUS ESTABLISHMENT MODEL

We use a two-locus establishment model to analyse the importance of the establishment advantage of mutations landing close to a diverged locus in comparison to those landing at a distance. In these simulations we assume that one of the two loci has diverged before a new mutation lands in the genome. This locus is assumed to have alleles of effect sizes $Y_{\mathrm{s}} > 0$ and $-Y_{\mathrm{s}}$ that are in a migration-selection balance. The initial frequencies of these two allele-effect sizes in the two populations are determined using a set of recursive deterministic equations (see **S4**). Note that in populations of infinite size, a balance between allele-effect sizes $Y_{\mathrm{s}}$ and $-Y_{\mathrm{s}}$ (both having nonzero frequencies, see **S4**) will be established and maintained under any migration rate due to the symmetries assumed in the model (Yeaman and Otto 2011) (but the allele frequencies depend on the migration rate). With these settings, the extent of local genomic divergence $D_{\mathrm{s}}$ at this locus prior to mutation is $D_{\mathrm{s}} = 4Y_{\mathrm{s}}$. In addition, the second locus is assumed to have alleles of effect size zero prior to mutation. Therefore the extent of local genomic divergence $D_{\mathrm{w}}$ at this locus is equal to zero. Thereafter we assume that a mutation lands on the undifferentiated locus (or the diverged one, see below) in the population where it is locally beneficial, and we simulate the dynamics of genotype frequencies under drift to estimate the probability that the mutation successfully establishes in the populations. In these simulations, the mutation-effect size $\epsilon > 0$ is assumed to be fixed and equal to the standard deviation $\sigma_\mu$ of the mutation-effect size distribution. Because the mutation of size $\epsilon > 0$ is beneficial in the first population (with the positive optimal phenotype) we assume it lands in the first population. Thereafter, new mutations are not allowed. We neglect mutations landing in the population where they are locally deleterious because these are much less likely to establish successfully in comparison to mutations landing in the population where they are locally favourable. Each simulation is advanced until either the mutant allele experiences extinction, or until it becomes most common (frequency $> 50\%$) at the locus in the population where it is beneficial. In the latter case, a successful establishment event is noted. We run at least $10^5$ such independent simulations, and the establishment probability is estimated as the proportion of successful establishment events among all independent runs made. We estimate the establishment probabilities of a new mutation landing at various recombination distances $r_j$ from the diverged locus. The values of $r_j$ are chosen as $r_j = jr$ ($j = 0, \ldots, 50$), where $r = 0.0005$ corresponds to the recombination distance between adjacent loci set in Fig. 1. When $j = 0$, a mutation lands on the diverged locus ('stacking' à la Yeaman and Whitlock (2011)). In this case, we additionally

assume that a mutation lands on a locally favourable allele, giving rise to the mutant allele that is locally advantageous in comparison to either allele at this locus prior to the mutation. When $j = 50$, the distance between the two loci corresponds to a half of the total recombination distance assumed in Fig. 1. Varying $Y_\mathrm{s}$ from zero to unity we approximate different stages of divergence from no divergence to perfect local adaptation in both populations. Results obtained in this model are shown in Figs. S2-S3.

## S3. TWO-LOCUS GAIN-LOSS MODEL

In this appendix we explain the assumptions used in the two-locus gain-loss model that is introduced in the main text. In this model the two loci are assumed to be at a recombination distance $r_j > 0$. Both loci are assumed to be differentiated initially, one with a stronger and the other with a weaker extent of divergence. Each locus has two alleles with effect sizes that are symmetric around zero ($Y_\mathrm{s} > 0$ and $-Y_\mathrm{s}$ at the more strongly diverged locus, and $0 < Y_\mathrm{w} < Y_\mathrm{s}$ and $-Y_\mathrm{w}$ at the weakly diverged one). We set the allele-effect sizes at the weakly diverged locus to $Y_\mathrm{w} = \sigma_\mu$, and $-Y_\mathrm{w}$. We choose this value as a representative beneficial mutation-effect size in the first population in a situation when mutation-effect sizes are drawn from a Gaussian distribution with a zero mean and a standard deviation $\sigma_\mu$ (assuming that when divergence starts all loci have alleles with effect sizes zero). Mutation-effect sizes much smaller than this value appear with a higher probability, but they suffer from a lower establishment probability. By contrast, mutation-effect sizes larger than this value appear with a much smaller probability. The initial haplotype frequencies are assumed to be equal to those in the deterministically expected stable steady state of the system (see **S4**). After initialisation we run two sets of simulations. In one we aim to estimate the rates of local loss at the two loci (neglecting new mutations). In the other we aim at estimating the rate of local gain upon introducing a mutation. These two sets of simulations are described in the main text, where we also show and discuss the results obtained under the gain-loss model.

## S4. DETERMINISTIC APPROXIMATION FOR A TWO-LOCUS MODEL

In this appendix we list a set of recursive two-locus deterministic equations for adaptive divergence that we use to determine haplotype frequencies at the start of simulations of the establishment, and gain-loss models introduced in **Materials and Methods** in the main text. The deter-

ministic approximation for the dynamics of haplotype frequencies is valid in the limit of infinitely large populations.

The main assumptions of the model of adaptive divergence are introduced in the main text. The populations are assumed to be diploid and of equal size $N$ that is constant over time. For purposes of this appendix, we assume here that $N \to \infty$. The environmental conditions are assumed to be different in the two demes, so that a given phenotype is under divergent selection. The optimal phenotype in the first (second) deme is denoted by $\theta^{(1)}$ ($\theta^{(2)}$), and we assume that $\theta^{(1)} > 0$, and $\theta^{(2)} = -\theta^{(1)}$. In the two-locus model, the phenotype of an individual is assumed to be determined by the diploid genotype at two adaptive loci. Each allele at a given locus is assigned an allele-effect size by which it additively contributes to the phenotype. Selection is assumed to be soft, so that a contribution of individual $i$ with phenotype $z$ to the gamete pool in deme $k$ is proportional to the fitness $w_i^{(k)}$ of this individual relative to the fitness of all individuals in this deme, where

$$w_i^{(k)} = \mathrm{e}^{-\frac{(z-\theta^{(k)})^2}{2\sigma^2}} \quad . \tag{S1}$$

The strength of selection is determined by the parameter $\sigma$ in such a manner that selection is weaker when $\sigma$ is larger, and vice versa. In the model, individuals firstly migrate to the opposite deme at a rate $m$ per individual, generation. Thereafter, random mating, recombination and selection occur locally within each deme. Recombination is assumed to occur at a rate $r$ per gamete, individual, generation. When $r = 0$, the model described corresponds to a single-locus model.

In this appendix we assume that each locus has two possible alleles, and we aim at estimating the haplotype frequencies in the steady state of the system. The effect sizes of these alleles are assumed to be symmetric around zero, and we denote them by $Y_\mathrm{s} > 0$ and $-Y_\mathrm{s}$ at one locus, and $Y_\mathrm{w} > 0$ and $-Y_\mathrm{w}$ at the other locus. We assume that $Y_\mathrm{s}$, and $Y_\mathrm{w}$ are advantageous over $-Y_\mathrm{s}$, and $-Y_\mathrm{w}$, respectively in the first population. The opposite is true in the second population. When $Y_\mathrm{s} = Y_\mathrm{w}$ the two loci do not differ in the extents of their divergence, whereas for $Y_\mathrm{s} > Y_\mathrm{w}$, the first locus has a higher extent of divergence than the second one. In what follows, we use a deterministic approximation to find the haplotype (and allele) frequencies at the two loci in the stable steady state.

When two divergent populations are initialised with allele-effect sizes $x_1 = Y_\mathrm{s}$ and $x_2 = -Y_\mathrm{s}$ at one locus, and with $y_1 = Y_\mathrm{w}$ and $y_2 = -Y_\mathrm{w}$ at the other locus, a deterministic approximation shows that each locus establishes a stable dimorphism (see also Yeaman and Otto (2011)).

This conclusion can be arrived at by iterating a system of recursive equations for the evolution of frequencies $p^{(k)}_{x_i,y_j;\tau}$, of haplotypes $x_i, y_j$ $(i,j = 1,2)$ in the two populations $(k = 1,2)$ from generation $\tau$ to generation $\tau+1$. The dynamics are fully determined by a set of six equations. For simplicity, however, we show here the corresponding equation for $p^{(1)}_{x_1,y_1;\tau+1}$ noting that the remaining five equations are obtained similarly:

$$
\begin{aligned}
p^{(1)}_{x_1,y_1;\tau+1} =& \Big[(1-m)\Big(p^{(1)}_{x_1,y_1;\tau}\Big)^2 + m\Big(p^{(2)}_{x_1,y_1;\tau}\Big)^2\Big]\frac{w^{(1)}_{x_1,y_1|x_1,y_1}}{\langle w^{(1)}_\tau\rangle} \\
&+ \Big[(1-m)p^{(1)}_{x_1,y_1;\tau}p^{(1)}_{x_1,y_2;\tau} + mp^{(2)}_{x_1,y_1;\tau}p^{(2)}_{x_1,y_2;\tau}\Big]\frac{w^{(1)}_{x_1,y_1|x_1,y_2}}{\langle w^{(1)}_\tau\rangle} \\
&+ \Big[(1-m)p^{(1)}_{x_1,y_1;\tau}p^{(1)}_{x_2,y_1;\tau} + mp^{(2)}_{x_1,y_1;\tau}p^{(2)}_{x_2,y_1;\tau}\Big]\frac{w^{(1)}_{x_1,y_1|x_2,y_1;\tau}}{\langle w^{(1)}_\tau\rangle} \\
&+ r\Big[(1-m)p^{(1)}_{x_1,y_2;\tau}p^{(1)}_{x_2,y_1;\tau} + mp^{(2)}_{x_1,y_2;\tau}p^{(2)}_{x_2,y_1;\tau}\Big]\frac{w^{(1)}_{x_1,y_2|x_2,y_1}}{\langle w^{(1)}_\tau\rangle} \\
&+ (1-r)\Big[(1-m)p^{(1)}_{x_1,y_1;\tau}p^{(1)}_{x_2,y_2;\tau} + mp^{(2)}_{x_1,y_1;\tau}p^{(2)}_{x_2,y_2;\tau}\Big]\frac{w^{(1)}_{x_1,y_1|x_2,y_2}}{\langle w^{(1)}_\tau\rangle}\ . \qquad \text{(S2)}
\end{aligned}
$$

Here, $\langle w^{(1)}_\tau\rangle$ denotes the average fitness of parents in the first population in generation $\tau$ and it is given by

$$
\langle w^{(1)}_\tau\rangle = \sum_{i=1}^{2}\sum_{j=1}^{2}\sum_{l=1}^{2}\sum_{a=1}^{2}\Big[(1-m)p^{(1)}_{x_i,y_j;\tau}p^{(1)}_{x_l,y_a;\tau} + mp^{(2)}_{x_i,y_j;\tau}p^{(2)}_{x_l,y_a;\tau}\Big]w^{(1)}_{x_i,y_j|x_l,y_a}\ , \qquad \text{(S3)}
$$

where the subscripts $i$ and $j$ denote alleles at the two loci at one chromosome (similarly, $l = 1,2$ and $a = 1,2$ are used for the corresponding pair at the other chromosome). The superscript $k = 1,2$ stands for the first and second population, respectively. The fitnesses $w^{(k)}_{x_i,y_j|x_l,y_a}$ $(i,j,k,l,a = 1,2)$ are given by Eq. (S1) with $z = x_i + y_j + x_l + y_a$.

Using Eq. (S2) and the remaining five equations of the system, we find recursively the state at which the system eventually relaxes within a predetermined numerical precision. As a stopping condition for finding this state we require that during $1000$ successive generations neither of the squared allelic frequencies change by more than $10^{-8}$. The maximum number of generations for finding the steady state is set to $10^5$. For all parameter values tested, we find a stable dimorphism with nonzero allele frequencies at both loci as well as linkage disequilibrium between loci, the

extent of which can be determined according to the haplotype frequencies obtained.

In a special case when $r = 0$, and the system is initialised with haplotypes $Y_\mathrm{s}, Y_\mathrm{w}$ and $-Y_\mathrm{s}, -Y_\mathrm{w}$ (effectively corresponding to allele-effect sizes $Y_\mathrm{s} + Y_\mathrm{w}$ and $-Y_\mathrm{s} - Y_\mathrm{w}$ at a single locus), a deterministic approximation shows that, independently of the migration rate, the stable steady state of the system corresponds to both alleles having nonzero frequencies (but the actual frequencies depend on the migration rate and the selective advantage of locally beneficial allele). This stems from the analysis performed in Yeaman and Otto (2011) upon assuming in their model symmetric migration, and that the alleles labelled by $A$, and $a$ have effect sizes $-Y_\mathrm{s} - Y_\mathrm{w}$, and $Y_\mathrm{s} + Y_\mathrm{w}$, respectively. A stable dimorphism in the single-locus case is a consequence of the symmetries assumed in the model. As mentioned already, allele frequencies in the stable state attained depend on the migration rate, so that the frequency of locally favourable alleles is smaller under stronger migration.

We finalise this appendix by noting that we use the equations given above only to estimate the initial haplotype (and hence allele) frequencies in simulations under the two-locus establishment model, and under the two-locus gain-loss model introduced in the main text. All simulations are otherwise stochastic and performed under random genetic drift.

## S5. REFERENCES


Feder, J. L., R. Gejji, S. Yeaman, and P. Nosil. 2012. Establishment of new mutations under divergence and genome hitchhiking. Philos. Trans. R. Soc. Lond. B Biol. Sci. 367(1587): 461–474.

Yeaman, S., and S. P. Otto. 2011. Establishment and maintenance of adaptive genetic divergence under migration, selection, and drift. Evolution 65(7): 2123–2129.

Yeaman, S. and M. C. Whitlock. 2011. The genetic architecture of adaptation under migration-selection balance. Evolution 65(7): 1897–1911.

## S6. SUPPLEMENTARY FIGURES

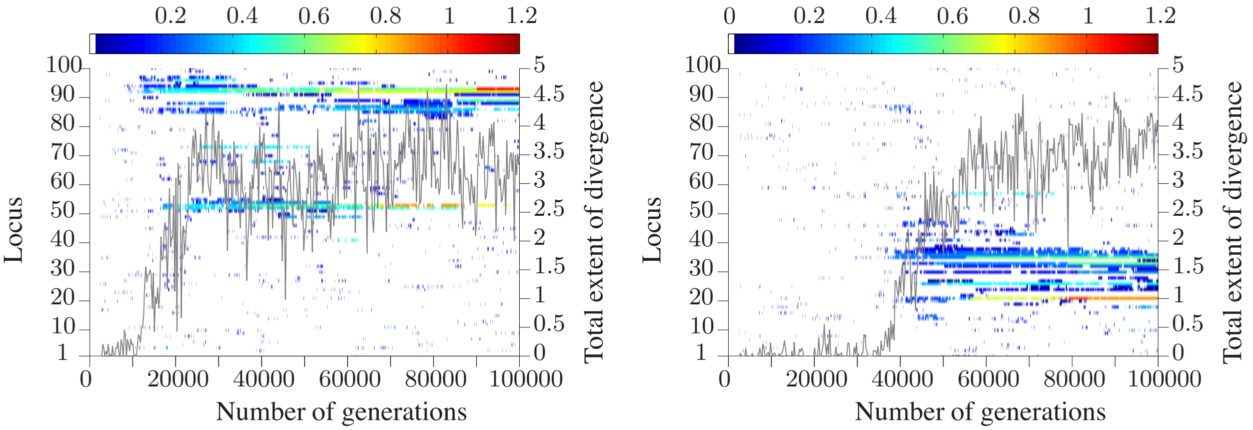


FIG. S1: Same as in Fig. 1**A** in the main text, but here patterns from two different realisations are shown. For clarity, grey lines here depict the total extent of divergence in intervals of 250 generations (whereas Fig. 1**A** shows all measures in intervals of 50 generations). For the explanation and parameter values used, refer to Fig. 1**A** in the main text.

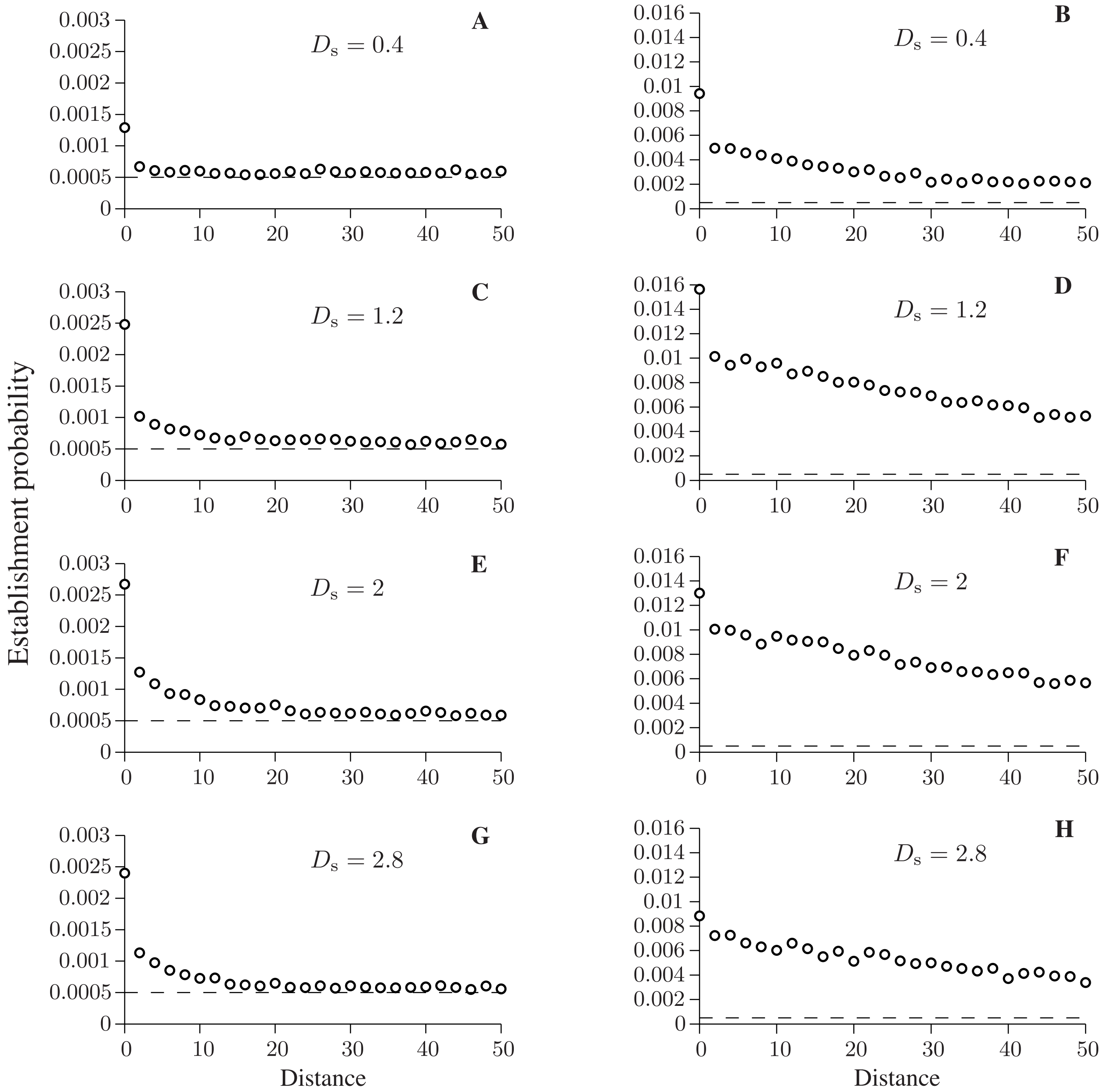


FIG. S2: Results of the two-locus establishment model. Shown is the probability of establishment of a new mutation of a fixed size landing at an undifferentiated locus as a function of the distance between this locus and the locus that is differentiated prior to the mutation (measured in units of the recombination rate $r$). The establishment probability at distance zero corresponds to the mutation landing at the already differentiated locus. Dashed lines show the probability $1/(2N)$ of fixation of a neutral mutation at a neutral locus in a diploid population of size $N$. Panels differ by the extent of divergence $D_s$ at the more strongly diverged locus prior to the mutation. Selection is weaker in **A**, **C**, **E**, **G** ($\sigma = 4$) than in **B**, **D**, **F**, **H** ($\sigma = 2.5$). Remaining parameter values: population size in each deme $N = 1000$, migration rate $m = 0.1$, recombination rate $r = 5 \cdot 10^{-4}$, mutation-effect size $\epsilon = 0.05$, $10^6$ independent simulations in **A**, **C**, **E**, **G**, and $10^5$ in **B**, **D**, **F**, **H**.

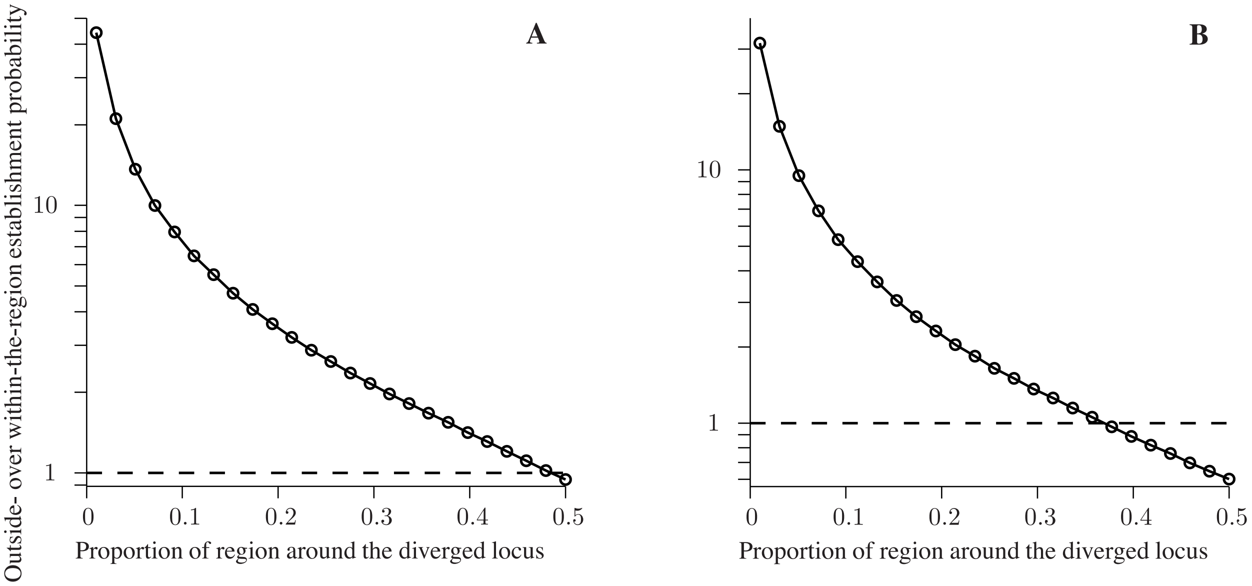


FIG. S3: Establishment bias in the two-locus establishment model. Shown is the integral of the establishment probability over distances outside of a given region around the diverged locus relative to the integral over distances within the region, as a function of the proportion that the region accounts for (out of $L = 100$ loci). Dashed line indicates the ratio of unity. Note that in **A** the ratio is, as expected, below unity (approximately $0.95$) when the proportion of the region around the diverged locus is equal to $0.5$, but this is difficult to observe due to the scale of the $y$-axis used. Selection is weaker in **A** ($\sigma = 4$) than in **B** ($\sigma = 2.5$). Parameters: mutation-effect size $\epsilon = 0.05$, the extent of divergence at the already diverged locus $D_{\mathrm{s}} = 0.4$, population size in each deme $N = 1000$, migration rate $m = 0.1$, $10^6$ independent simulations in **A**, and $10^5$ in **B**.

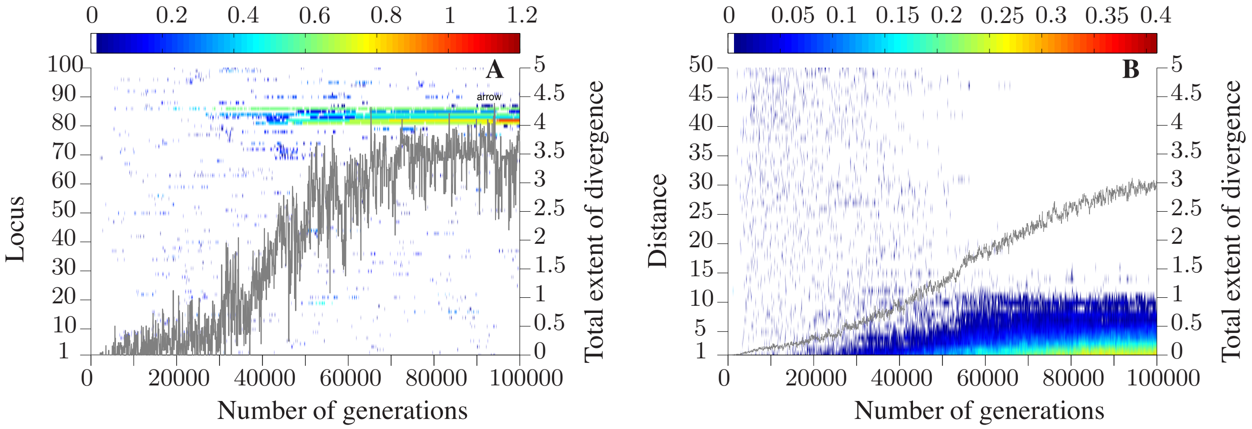


FIG. S4: Patterns of divergence under the parameter values similar to those in Fig. 1**A**, **B** in the main text, but here the recombination distance between adjacent loci is two times larger ($r = 0.001$). Shown are the results from a single realisation in **A**, and averages over $54$ independent realisations in **B**. Remaining parameter values are the same as in Fig. 1**A** in the main text.

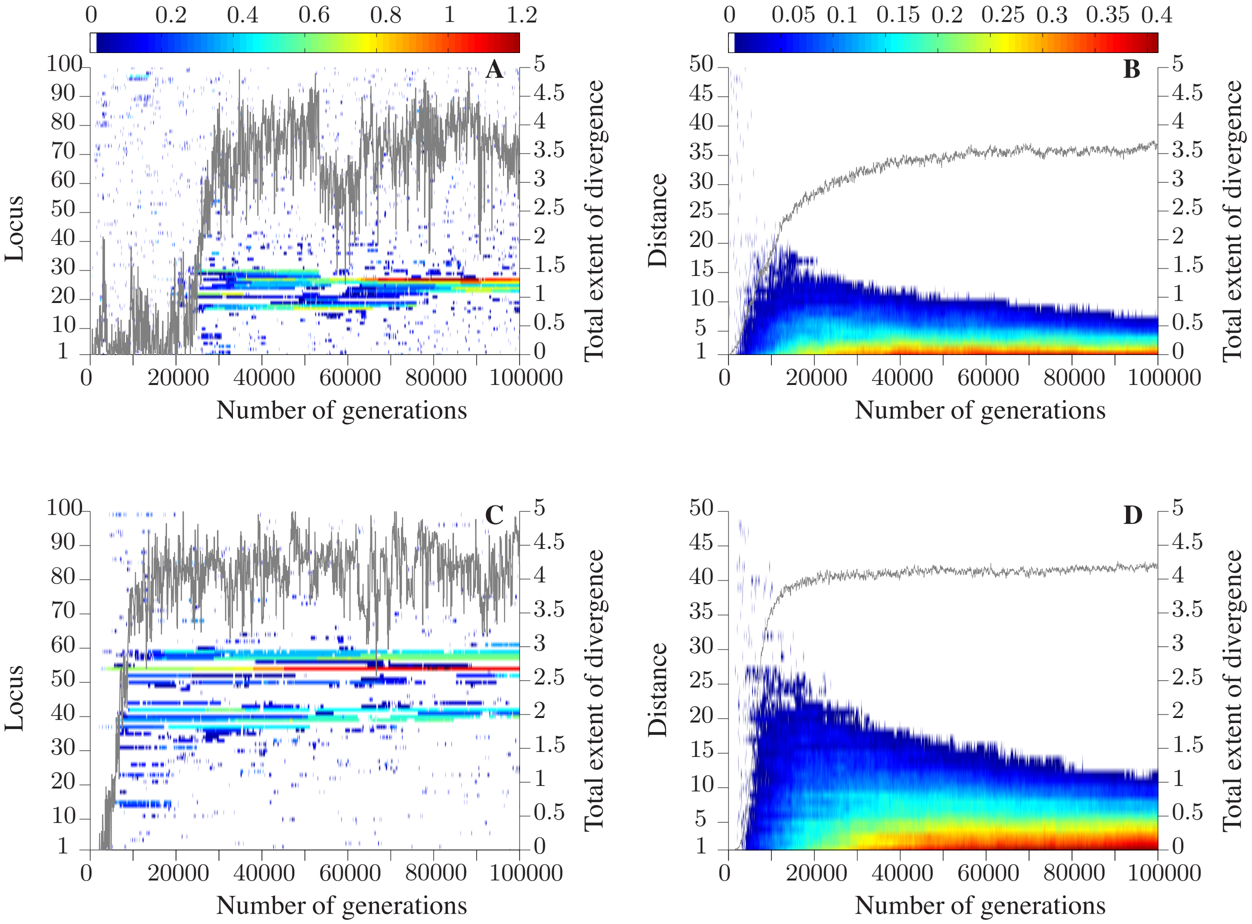


FIG. S5: Effect of drift. Same as in Fig. 1 in the main text, but here we contrast the results obtained under a small population size ($N = 200$, **A**, **B**), and a large population size ($N = 1000$, **C**, **D**). The mutation rate is set so that its value scaled by the corresponding population size is equal in the two cases ($\mu = 10^{-4}$ in **A** and **B**, or $\mu = 2 \cdot 10^{-5}$ in **C** and **D**). In both cases, the selection parameter is $\sigma = 3.5$. Other parameter values are the same as in Fig. 1 in the main text.

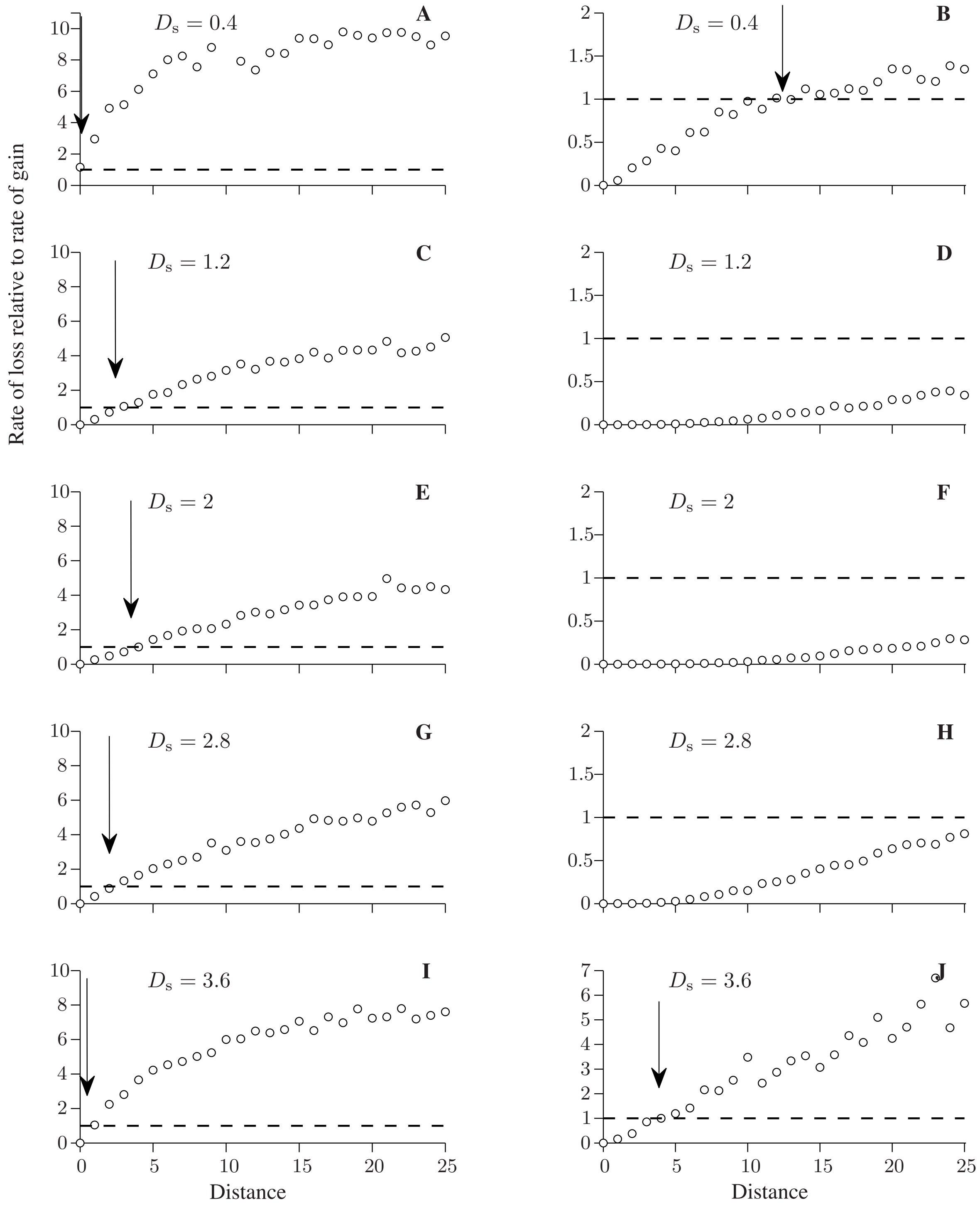


FIG. S6: Same as in Fig. 3 in the main text but for the selection parameter $\sigma = 3.5$ (corresponding to that used in Fig. S5). For the explanation of the figure refer to Fig. 3 in the main text. Panels differ by the initial extent of divergence $D_s$ at the more strongly diverged locus. Population size: $N = 200$ (in **A**, **C**, **E**, **G** and **I**), and $N = 1000$ (in **B**, **D**, **F**, **H** and **J**). Mutation rate: $\mu = 10^{-4}$ (in **A**, **C**, **E**, **G** and **I**), and $2 \cdot 10^{-5}$ (in **B**, **D**, **F**, **H** and **J**). Mutation-effect size: $\epsilon = 0.05$. For each parameter combination, the rate of gain is estimated based on $10^5$ independent simulations. The rate of loss is estimated using $10^3$ independent simulations (in **A**, **C**, **E**, **G**, **I**), or 500 simulations (in **B**, **D**, **F**, **H**, **J**). Other parameters are the same as in Fig. S5.

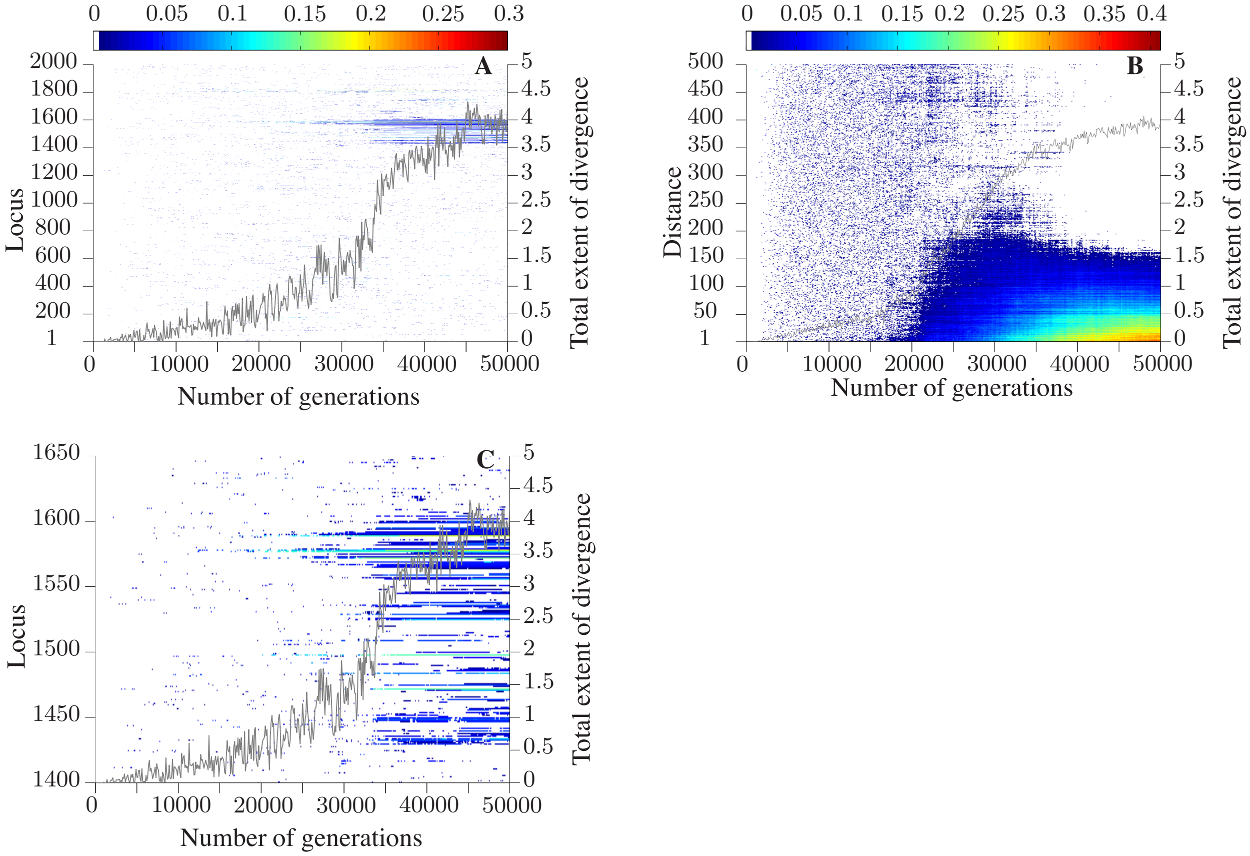


FIG. S7: Patterns of divergence under the parameter values similar to those in Fig. 1**C** in the main text but with 20 times more adaptive loci ($L = 2000$), and 20 times smaller variance $\sigma_\mu^2$ of mutation-effect sizes. Panel **A**: the extent of divergence at all loci in a single stochastic realisation of the model. The solid line shows the total extent of divergence in the underlying single realisation of the model (the values are depicted on the $y$-axis on the right). Panel **B**: correlations at pairs of loci as a function of time and the distance between them (measured in units of recombination rate $r$) averaged over 10 independent realisations. The corresponding average total extent of divergence is shown by the solid line. Panel **C**: same as in **A**, but depicting a cluster of diverged loci that accounts for most of the total extent of divergence in the realisation shown. Loci are assumed to reside on two chromosomes, so that the loci labelled $1, \ldots, 1000$ are on one chromosome, and loci labelled $1001, \ldots, 2000$ are on the other. Root mean square of mutation-effect sizes: $\sigma_\mu = 0.05/\sqrt{20}$. Remaining parameters are the same as in Fig. 1**C**.

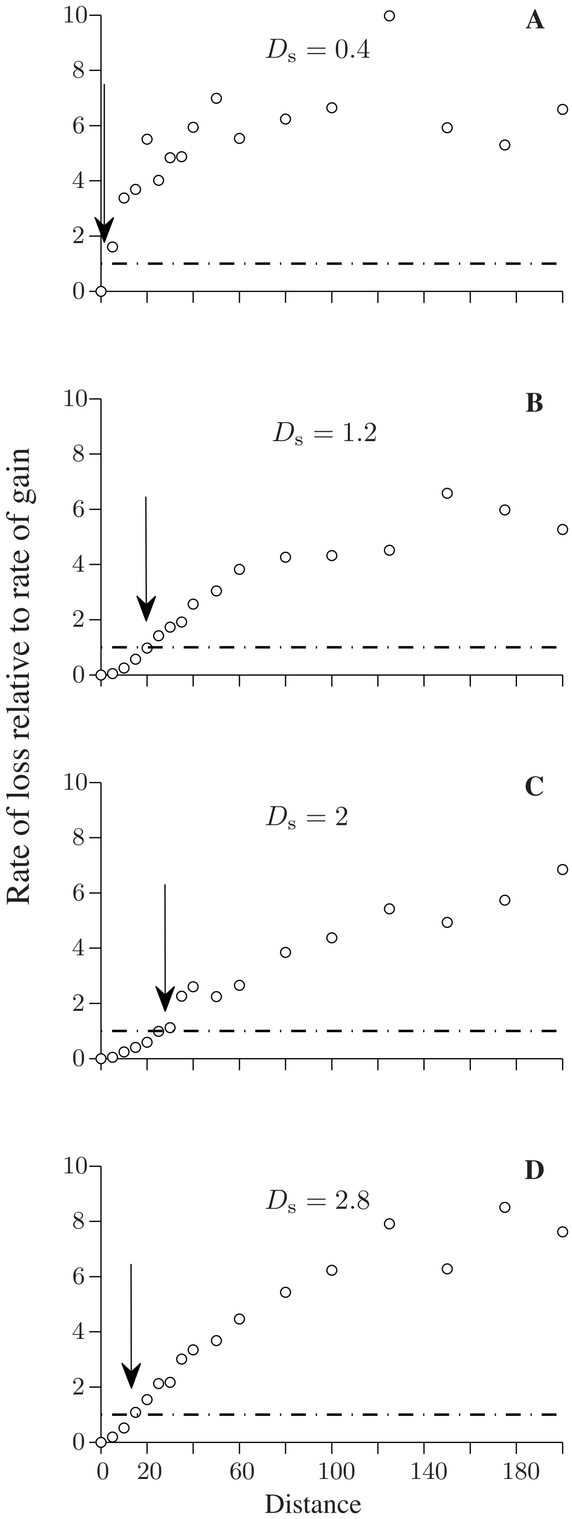


FIG. S8: Same as in Fig. 3 in the main text but for the parameters corresponding to those in Fig. S7. Panels differ by the initial extent of divergence $D_{\rm s}$ at the more strongly diverged locus. Mutation-effect size: $\epsilon = 0.05/\sqrt{20}$. The extent of divergence $D_{\rm w}$ at the weakly diverged locus is set to $D_{\rm w} = 4\epsilon$ (i. e. $D_{\rm w} = 0.2/\sqrt{20}$). The rate of gain is estimated using $10^5$ independent simulations. The rate of loss is based on 200 simulations. Other parameters are the same as in Fig. S7.

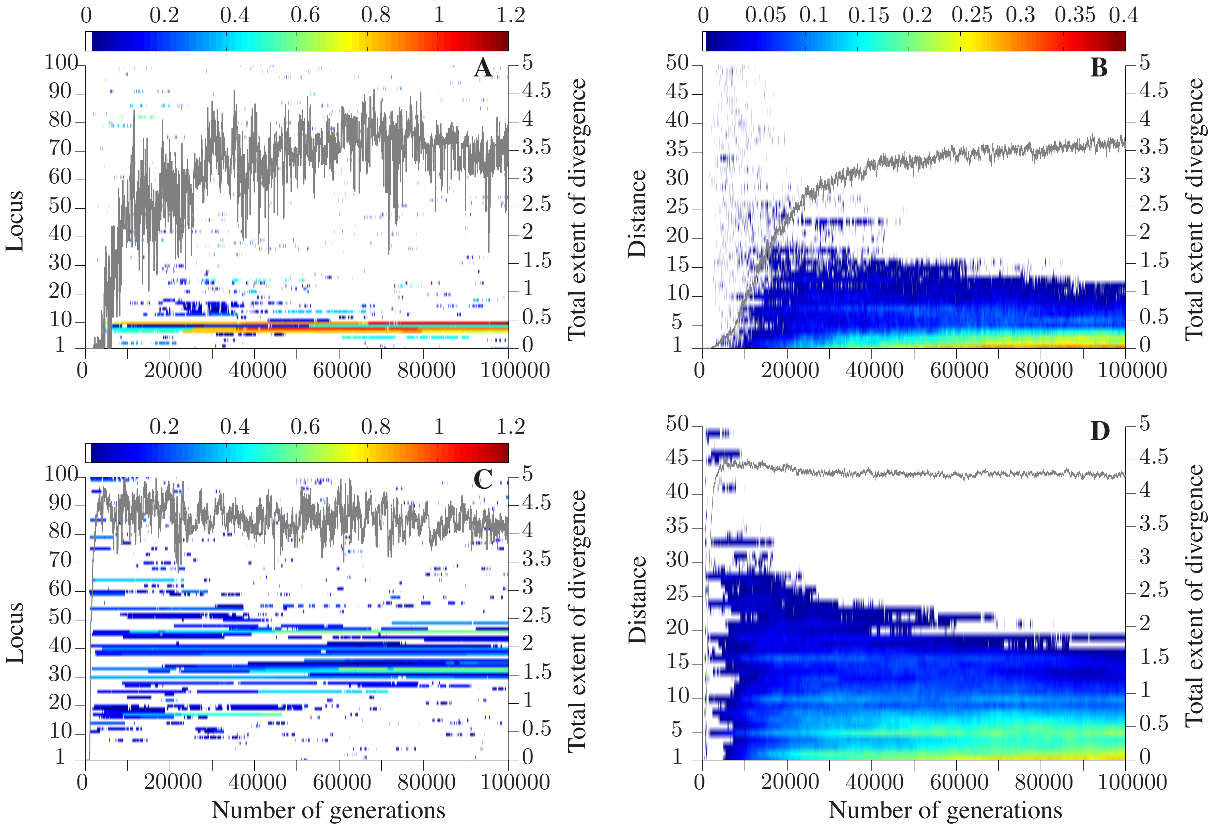


FIG. S9: Patterns of divergence for the parameter values corresponding to those in Figure 1 in the main text, but here mutation-effect sizes are drawn from an exponential distribution mirrored around zero (that is, positive and negative effects are assumed to be equally likely). For the explanation of the results shown refer to the caption of Figure 1 in the main text. Number of independent realisations in panels **B**, **D**: 50. Remaining parameter values are the same as in Fig. 1 in the main text.

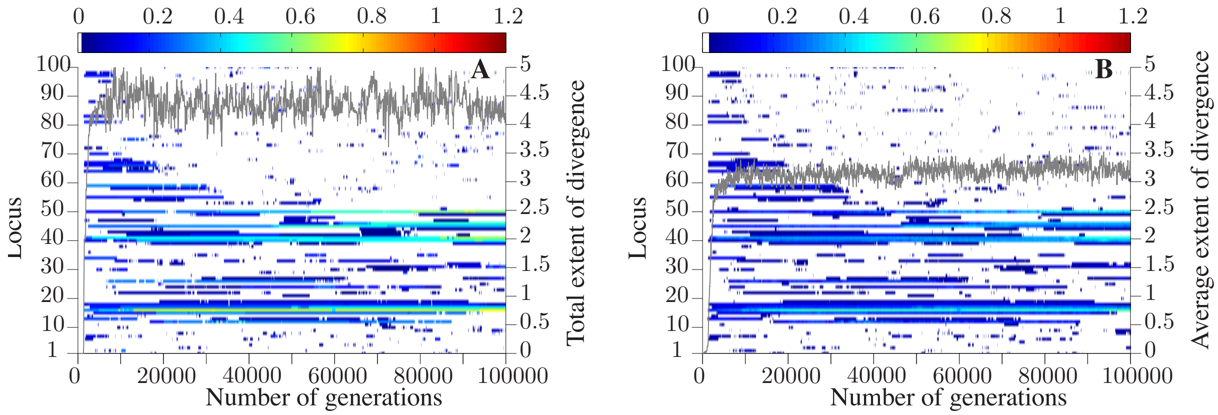


FIG. S10: A comparison between patterns of divergence in a single stochastic realisation of the model, but shown using two different measures for the extent of divergence at locus $j$, that is, in **A** we use the measure $D_l$ introduced in the main text (the total extent of divergence is equal to $\sum_{l=1}^{L} D_l$), and in **B** we use instead twice the difference between average allele-effect sizes at locus $l$ in the two populations (the average extent of divergence is equal to the sum of average allele-effect sizes at all $L$ loci simulated). All parameter values correspond to those in Figure 1**C** in the main text, but here the result of a different stochastic realisation is shown. For further explanation of the results shown refer to the caption of Figure 1**C** in the main text.
